# Sickness absence in the working age population: a retrospective cohort study using primary care health record data

**DOI:** 10.1186/s12889-026-26296-6

**Published:** 2026-02-05

**Authors:** Naijie Guan, James Rockey, Tom Marshall, Eleanor Hathaway, Tracy Roberts, Francesca Crowe, Louise J. Jackson, Shamil Haroon

**Affiliations:** 1 Department of Applied Health Sciences, University of Birmingham, Birmingham, UK; 2 Department of Economics, University of Birmingham, Birmingham, UK

## Abstract

**Background:**

Economic inactivity rate in the UK reached 22.2% in 2024, driven largely by increased long-term sickness, and exceeds those reported in comparable high-income countries. Sickness absence remains a major challenge, imposing substantial costs on individuals and society. This study aimed to assess sickness absence rates and associated economic output losses in primary care for working-age adults in England.

**Methods:**

This was a population-based retrospective cohort study using primary care data from the Clinical Practice Research Datalink (CPRD) Aurum database. 10 million adults aged 18–65 years was followed from January 2017 to December 2019 (pre-COVID-19 pandemic period) and March 2022 to February 2023 (late pandemic period). Sickness absence was measured using fit notes (“statements of fitness for work”) issued in primary care. We quantified annual fit note rates, identified underlying medical causes, explored associations between fit note provision, patient’s sociodemographic and health-related factors using random-effect negative binomial regression models, and estimated economic losses due to sickness absence.

**Results:**

Age-standardised fit note rates were 23.5 per 100 person-years pre-pandemic and 24.0 per 100 person-years in the late pandemic period. Fit notes were issued more frequently among females, older individuals, black ethnic groups, lower-income groups, those with obesity, smokers, and those with higher levels of comorbidity. Depression and anxiety were the leading medical diagnoses associated with fit note provision. The estimated total annual cost of sickness absence in England was around £13.3 billion (95% CI £13.3 to £13.4 bn) in the pre-pandemic period, and £15.9 bn (95% CI £15.8 to £15.9 bn) in the late pandemic period.

**Conclusions:**

Fit note provision in primary care and associated costs rose substantially from the pre-pandemic to the late pandemic period. This was primarily associated with poor mental health and varied among different population groups. Towards the end of the pandemic, this represented approximately £16 billion in annual economic losses in England. This underscores the need for targeted policy interventions and further research to mitigate the health and financial burden of sickness absence, particularly for people with poor mental health.

**Supplementary Information:**

The online version contains supplementary material available at 10.1186/s12889-026-26296-6.

## Introduction

Despite various economic indicators returning to pre-pandemic levels, the UK employment rate remains below its pre-pandemic level, unlike in other G7 economies [1, 2]. This is not due to rising unemployment, which remains low despite a recent uptick, but rather due to an alarming increase in economic inactivity [2]. Economic inactivity refers to working-age adults who are not part of the labour market. Data for the quarter from December 2023 to February 2024 show that the economic inactivity rate has surged to 22.2%, representing 9.4 million people, the highest level since 2015 [1, 2]. This represents a stark reversal from the 25 years preceding the COVID-19 pandemic, during which there were consistent improvements in labour force participation [1, 2]. A major driver of increased economic inactivity is the rise in long-term sickness, which now affects a record 2.8 million people and accounts for over 30% of the economically inactive population [3–5].

Furthermore, even among those who remain in the workforce, sickness absence has increased in recent years, both in the UK and across Europe [6–8]. Sickness absence refers to absence from work due to ill health that affects an individual's ability to work, encompassing both medical, psychological, social, and economic aspects [6]. It is commonly seen as an indicator of the health status of the working population in a society [6]. According to estimates from the UK Office for National Statistics [9], the sickness absence rate reached 2.6% in 2022, the highest since 2004, when it was 2.7% [9]. The rise in sickness absence is not unique to the UK; similar upward trends have been observed in many European countries. According to the Eurostat’s statistics, sickness absence across Europe peaked in 2022 [7, 8]. It remained elevated in 2024, with around two-thirds of EU countries reporting higher sickness absence than before the pandemic [7, 8].

Evidence has also shown that sickness absences vary across different demographic and socioeconomic groups [10]. Specifically, factors such as age, gender, socioeconomic status, and job satisfaction can affect patterns of sickness absence [9]. Furthermore, different GP practices and healthcare professional types may have varying thresholds and considerations when making medical decisions related to issuing fit notes or statements for fitness to work (also referred to as sick notes) [11, 12].

Sickness absence imposes substantial direct and indirect costs on employees and employers [13]. In 2022, a record 185.6 million working days were lost due to sickness or injury, indicating significant socioeconomic impacts [14]. The UK’s Office for Budget Responsibility estimated that health-related economic inactivity has cost the government £15.7 billion annually since the COVID-19 pandemic began, representing both a loss of tax revenue and increased welfare spending [15]. At the individual level, the Institute of Public Policy Research estimated that the onset of a chronic health condition can substantially reduce an individual's earnings. During the five years preceding the pandemic, approximately one-third of individuals with a new onset of a chronic illness, around 2.1 million people, experienced a 10% or greater decline in earnings [16].

In the UK, individuals certify themselves as unfit for work due to sickness for the first seven days. Medical certificates, also called “fit notes” or “sick notes”, are issued, usually by General Practitioners (GPs), to indicate an inability to work after seven days of sickness absence [13]. They were introduced in the UK in 2010 to support statutory sick pay for employees and to provide medical evidence for benefit claims, including for the self-employed [17]. Fit notes certify individuals as “not fit for work” or “may be fit for work” with potential adjustments [17]. However, over 90% of fit notes issued each year certify individuals as “not fit for work” [18], which may represent missed opportunities to support people in remaining in work with appropriate adjustments.

A fit note can be issued for up to three months at a time during the first six months of a health condition [19]. While consecutive fit notes may cover more extended periods, individuals whose health condition limits their ability to work for more than 28 days may be referred for a Work Capability Assessment (WCA) to determine eligibility for Employment and Support Allowance (ESA) or Universal Credit [20]. After a WCA concludes that a person has limited capability for work, ongoing fit-note certification is generally no longer required, as benefit entitlement is then based on the WCA decision rather than repeat fit notes.

In the UK, the issuance of fit notes rose notably from 8.7 million in the 12 months leading to March 2021 to 11 million in the same period ending March 2023 [21]. This underscores the importance of understanding more about the issuance of fit notes, the changing trends in fit note provision, and the associated health conditions, as they provide critical insights into the general health and well-being of the working-age population and its economic implications. A limited number of research studies have explored individual and societal factors associated with sickness absence [12, 22]. Those studies either focused on non-UK populations [22, 23], specific subpopulations (e.g., healthcare employees [10]), or used only pre-pandemic data [14, 24]. There is therefore a need for further research in this field to better inform health and employment policies in the UK and similar economies.

In this study, we used an extensive longitudinal database of primary care health records in England to assess sickness absence rates and associated economic output losses before and during the latter part of the COVID-19 pandemic among the working-age population.

## Methods

### Study design

This is a retrospective cohort study using data from electronic primary care records in England. Working-age adults were followed up from three years before the COVID-19 pandemic to one year during the latter part of the pandemic. The rates of fit notes provided during the two follow-up periods were quantified, and their associations with sociodemographic and health-related factors were assessed. We also estimated the economic losses in terms of the total value of days not worked associated with these sickness absences.

### Data source

Data were obtained from the Clinical Practice Research Datalink (CPRD) Aurum database, which contains anonymised routinely collected data from UK general practices using the EMIS Web® patient record system [25]. In June 2021, it included over 13 million actively registered patients, covering approximately 20% of the UK population and 15% of all UK general practices [26]. General practice registration is near-universal in England: as of 1 November 2025, 63.9 million individuals were registered with a GP, exceeding the 2024 mid-year population estimate of 58.6 million, indicating that GP registers capture almost the entire resident population [27, 28]. The CPRD Aurum database is broadly representative of the UK population and captures data including patient demographics, diagnoses, symptoms and prescriptions. Data extraction was performed using the Data Extraction for Epidemiological Research (DExtER) tool for automated clinical epidemiological studies [29]. Area-level deprivation data, the Index of Multiple (IMD) income decile, were linked and provided by CPRD.

### Study population

The study focuses on two cohorts of patients aged 18 to 65 years registered with general practices for more than 12 months before the study start date. The first cohort comprises patients followed up during the pre-pandemic period from 1 January 2017 to 31 December 2019, while the second cohort was followed up during the latter part of the pandemic from 1 March 2022 to 28 February 2023 (see Additional files 1 and 2). Patients were eligible if they were aged 18 to 65 years on the cohort start date and had been registered with a general practice for at least 12 months.

On 5 May 2023, the World Health Organisation announced that COVID-19 is no longer classified as a Public Health Emergency of International Concern [30]. Thus, we defined the time from 1 March 2022 to 28 February 2023 as the latter part of the COVID-19 pandemic. The data from 1 January 2020 to 28 February 2022 were excluded because the issuing of fit notes in primary care was likely to be affected by employment and public health policies such as furlough, self-isolation and shielding implemented in response to the COVID-19 pandemic [31].

### Study endpoint

Patients were followed up until the earliest of the following dates: death, the patient left the practice/dataset, the patient reached the age of 65 years, the practice ceased contributing to the database, or 31 December 2019 for the pre-pandemic cohort and 28 February 2023 for the late pandemic cohort.

### Covariates

We examined the demographic and social factors associated with the frequency of fit notes provided using regression models, which included the following covariates: age, sex, ethnicity, socioeconomic status, body mass index (BMI), smoking status, the number of prior consultations, comorbidities, general practice, and region (see Additional file 7).

Age was categorised into nine groups: 18–30 (reference group), 31–40, 41–50, 51–60, and 61–65. Sex was classified into two groups: females (reference group) and males. Ethnicity was categorised into six groups: White, Black, Asian, Mixed, Other, and Unknown. BMI was categorised into four groups: underweight (BMI < 18.5), normal weight (18.5—24.9), overweight (25—29.9), and obesity (≥ 30). Patients’ socioeconomic status was measured using the Index of Multiple Deprivation (IMD) income domain deciles, linked to the primary care dataset by CPRD. Practice regions were divided into nine areas: London, East Midlands, East of England, Northeast, Northwest, Southeast, Southwest, West Midlands, Yorkshire and The Humber. Smoking status was classified into four groups: never smoked, ex-smoker, current smoker, and missing. Comorbidities were represented using the Cambridge multimorbidity index, which calculates a weighted average of 21 health conditions [32] (see Additional file 8).

## Statistical analysis

### Descriptive statistics

Descriptive statistical analysis was conducted for the pre-pandemic and late-pandemic cohorts. For patient characteristics, categorical data were reported as frequencies and percentages, while continuous data were provided as means and standard deviations. For variables with missing values, we presented numbers and percentages of records with missing data.

### Fit note rates

Age-standardised overall and subgroup-specific (age, sex, ethnicity, income and region) incidence rates of fit notes issued were calculated for eligible patients during two periods: 1 January 2017 to 31 December 2019 and 1 March 2022 to 28 February 2023. Age standardisation was conducted using the age structure of the patients in CPRD Aurum in 2017 as the reference population. Medical codes used for fit notes are displayed in Additional file 19.

### Regression analysis

Multivariable random-effects negative binomial regression models were used to estimate the associations between fit note provision (the outcome variable) and sociodemographic and health-related factors described in the covariates section (explanatory variables). Negative binomial regression was selected over Poisson regression because the outcome variable was count data and exhibited overdispersion, as indicated by the alpha test (alpha, P < 0.001). This approach accounts for additional variability in the outcome variable that is not captured by Poisson regression.

A random-effects specification was used to account for clustering at the general practice level. This aims to account for unobserved heterogeneity across practices and adjusts for the intra-practice correlation of observations and unobserved heterogeneity between practices. For example, practices may differ in administrative processes, thresholds for issuing fit notes, or local implementation of policy, leading to correlated outcomes among patients within the same practice. Accounting for this correlation helps prevent biased standard errors and incorrect inference that may arise from ignoring such clustering. As a sensitivity analysis, multivariable fixed-effects negative binomial regression models were used to estimate the associations between fit note provision and sociodemographic and health-related factors. The sensitivity analysis was conducted to assess whether the results remained robust when fully controlling for time-invariant, unobserved practice-level characteristics.

Both adjusted incidence rate ratios (IRRs) and their 95% confidence intervals (CIs) were reported (Additional files 9 and 11). We also reported marginal effects from negative binomial regression models, which are displayed in Additional files 10 and 12. Analyses were stratified by study period (2017–2019 and 2022–2023) to assess temporal differences in these associations.

### Medical reasons for fit notes 

Each fit note was assigned a diagnostic category based on the most recent SNOMED-CT code recorded within a month before and on the date of fit note provision (and seven days prior to the fit note date in a sensitivity analysis). We focused on medical diagnoses that were the top contributors to years lived with disability (YLDs) for working-age individuals in England. They were identified using 2022 YLD rankings from the most recent Global Burden of Disease (GBD) study estimates available at the time of analysis [33]. The medical diagnoses selected include the most burdensome conditions contributing to YLDs, including major depression, anxiety, back pain, neck pain, knee osteoarthritis, migraine, alcohol misuse, asthma, chronic obstructive pulmonary disease (COPD), type 2 diabetes, falls, bipolar disorder, and hearing loss. We reported the frequency and proportion of all fit notes issued attributed to each of these diagnoses.

### Cost estimation

The cost of sickness absence was estimated in two steps. First, the expected duration of sickness absence was estimated by multiplying the number of fit notes issued by the expected duration of each fit note. However, data on the expected duration of each fit note is unavailable for over 95% of patients in CPRD Aurum. We therefore estimated this for the working-age population in England using publicly available fit note statistics published by NHS Digital [31]. These data classify fit notes by diagnostic group and duration (e.g., 1–7 days, 8–14 days, 15–28 days) and are stratified by calendar time, allowing us to estimate the average duration within each study year [31]. Additionally, we accounted for economic losses associated with the initial 7 days of self-certification, as fit notes are only issued after this period.

Second, economic losses associated with each sickness absence were estimated using area-level income data from the IMD income domain decile and IMD- and region-specific income estimates reported by ONS [34]. The economic loss due to sickness absence was calculated using the expected duration of sickness absence (in days) and the estimated average daily income loss. All cost estimates were adjusted for inflation and standardised to 2018 price levels. Further methodological details for the cost estimation are reported in Additional file 3. To assess the robustness of these estimates, a sensitivity analysis was conducted using IMD- and Lower Super Output Area (LSOA)-specific income estimates, which allow for more granular variation in local income distributions (also see Additional files 3, 15, and 16).

Statistical analyses were performed using Stata version 18, and figures were produced using R 4.5.0. A statement on patient and public involvement is available in Additional file 20.

## Results

### Study population characteristics

The characteristics of the study population are reported in Table 1. There were 11,406,264 and 10,044,331 individuals in cohorts 2017–2019 and 2022, respectively. Approximately 50.9–51.6% of the study population were male and 49.1–48.4% female. The mean age was 39.1 years (SD: 13.46) in the pre-pandemic cohort and 40.6 years (13.36) in 2022. About 69.5–69.6% of the study population were White, 9.7–11.5% were Asian, and 4.2–4.7% were Black (with the proportion of ethnic category coding improving over the course of the study period). One third of patients had a normal BMI, a quarter were overweight, and 17.7–19.4% were obese, with one fifth having missing BMI data. Just over 40% of the study population had never smoked, just under a quarter were ex-smokers, and just over a quarter were current smokers, with 6.6–8.8% having missing smoking status data. The study population included representation from all English regions.

**Table 1:** Characteristics of the study population in each cohort

Variables	Total individuals in 2017–2019 cohort N (%)	Total individuals 2022 cohort N (%)
**Sex**		
Male	5,799,628 (50.85)	5,181, 453 (51.59)
Female	5,606,636 (49.15)	4,862, 878 (48.41)
**Age**	Mean (SD):	Mean (SD)
	39.11 (13.46)	40.59 (13.36)
**Age group**		
18–30	3,539,226 (31.03)	2,705,371 (26.93)
31–40	2,598,419 (22.78)	2,356,901 (23.46)
41–50	2,303,210 (20.19)	2,056,229 (20.47)
51–60	2,141,001 (18.77)	2,066,582 (20.57)
61–65	824,408 (7.23)	859,248 (8.55)
**Ethnicity**		
White	7,923,389 (69.47)	6,987,029 (69.56)
Asian	1,107,689 (9.71)	1,156,854 (11.52)
Black	475,730 (4.17)	469,450 (4.67)
Other	193,780 (1.70)	205,896 (2.05)
Mixed	133,024 (1.17)	151,964 (1.51)
Missing	1,572,652 (13.79)	1,073,138 (10.68)
**BMI category**		
Underweight (< 18.5)	366,924 (3.22)	338,778 (3.37)
Normal (18.5–24.9)	3,865,864 (33.89)	3,260,809 (32.46)
Overweight (25–29.9)	2,801,032 (24.56)	2,507,540 (24.96)
Obese (> = 30)	2,018,738 (17.70)	1,952,265 (19.44)
Missing	2,353,706 (20.64)	1,984,939 (19.76)
**Smoking status**		
Never smoked	4,815,917 (42.22)	3,981,584 (39.64)
Ex-smoker	2,753,600 (24.14)	2,730,576 (27.19)
Current smoker	3,084,952 (27.05)	2,452,913 (24.42)
Missing	751,795 (6.59)	879,258 (8.75)
**Region**		
East Midlands	304,328 (2.67)	224,166 (2.23)
East of England	423,678 (3.71)	349,274 (3.48)
London	2,381,623 (20.88)	2,234,247 (22.24)
Northeast	418,151 (3.67)	360,885 (3.59)
Northwest	2,032,511 (17.82)	1,847,976 (18.40)
Southeast	2,472,976 (21.68)	2,160,702 (21.51)
Southwest	1,159,777 (10.17)	987,504 (9.83)
West Midlands	1,771,885 (15.53)	1,592,313 (15.85)
Yorkshire and the Humber	399,593 (3.50)	287,264 (2.86)
**Total number of patients**	***N* = 11,406,264**	***N* = 10,044,331**

### Fit note rates

The overall number of fit notes provided in the study period was 6,129,720 over 26,083,384 person-years (2,070,217 over 8,675,095 person-years in the late-pandemic period) of follow-up. Additional file 6 displays both crude and age-standardised fit note rates for the 2017, 2018, 2019, and 2022 cohorts, as well as rates across different population subgroups (i.e., sex, ethnicity, BMI, smoking status, and region). In 2017, the fit note rate was 23.6 per 100 person-years (95% CI 23.5 to 23.6). The age-standardised fit note rate per 100 person-years was 23.4 (95% CI 23.4 to 23.4) in 2018, 23.6 (95% CI 23.5 to 23.6) in 2019, and 24.0 (95% CI 24.0 to 24.0) in 2022, respectively.

Both crude and age-standardised fit note rates were higher in females than males in all cohorts. The crude and age-standardised fit note rates were higher among Black and White ethnic groups than among Asian and Mixed ethnicity groups. Crude and age-standardised fit note rates were highest in obese individuals, followed by overweight and underweight individuals, and lowest in those with a normal BMI. The crude and age-standardised fit note rates were higher in current and ex-smokers than never smokers. There was variation in the age-standardised fit note rates observed in different regions of England, with the lowest rates seen in London and East of England, and the highest in the Northeast and Northwest.

### Regression results

**Fig. 1:**
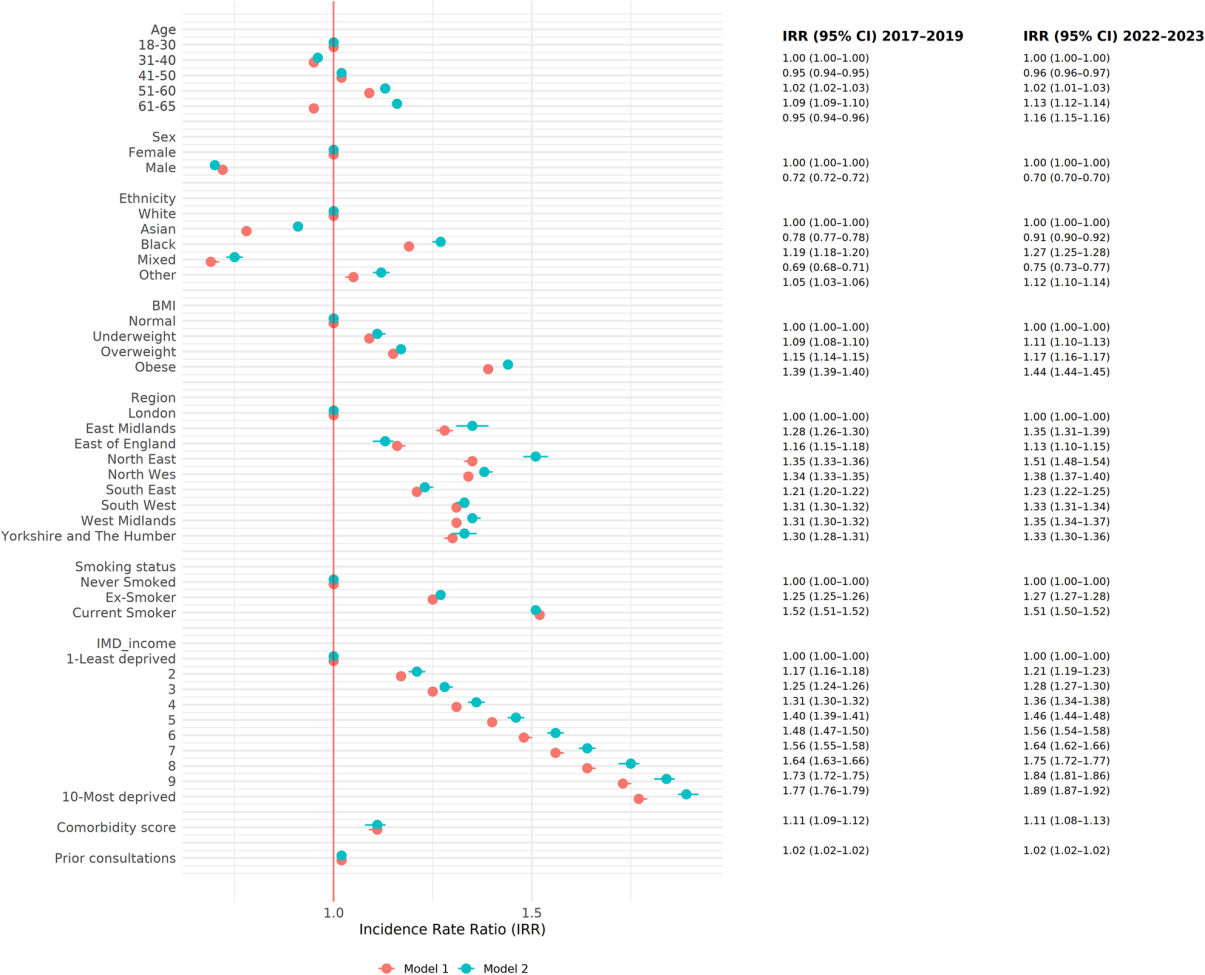
Associations between risk factors and fit note provision: incidence rate ratios (IRRs) with corresponding 95% confidence intervals from negative binomial regression analyses in the pre-pandemic cohort and the 2022 cohort. Notes: Model 1 estimates correspond to the pre-pandemic cohort, and Model 2 estimates correspond to the 2022 cohort. All estimates were adjusted for all covariates, including age, sex, ethnicity, body mass index (BMI), region, smoking status, the income domain of the Index of Multiple Deprivation (IMD), comorbidity index measured using the Cambridge Multimorbidity Score, number of prior consultations, and practice-level variation

Figure 1 and Additional file 9 present the incidence rate ratios (IRR) from random-effect negative binomial regression models for the provision of fit notes in the pre-COVID-19 cohort. In the adjusted models, males had a significantly lower fit note incidence rate than females, with an IRR of 0.72 (95% CI 0.72 to 0.72). Compared with individuals aged 18 to 30, those aged 41 and over had significantly higher fit note incidence rates. Individuals aged between 51 and 60 had the highest IRR when compared to those who were aged between 18 and 30 (IRR 1.09, 95% CI 1.09 to 1.10).

There were also significant ethnic disparities in the fit note incidence rates, where individuals from Black ethnic minority groups had a higher incidence rate (IRR 1.19, 95% CI 1.18 to 1.20) than those from White ethnic groups, while Asian and individuals from Mixed ethnic backgrounds had lower rates (IRR 0.78, 95% CI 0.77 to 0.78; and IRR 0.69, 95% CI 0.68 to 0.71, respectively). There was a clear gradient in the association between income deprivation and the outcome of interest, with individuals from more deprived areas experiencing a significantly higher risk than those from the least deprived group. Compared to individuals in the least deprived decile, those in the most deprived decile had a greater than 70% increased relative risk of being issued a fit note, with an IRR of 1.77 (95% CI 1.76 to 1.79).

The models also showed that obesity was associated with a significantly increased fit note incidence rate (IRR 1.39, 95% CI 1.39 to 1.40) when compared to individuals with a normal BMI. Similarly, both current (IRR 1.52, 95% CI 1.51 to 1.52) and ex-smokers (IRR 1.25, 95% CI 1.25 to 1.26) had a significantly higher fit note incidence rate than never smokers. Higher comorbidity index was also associated with a greater probability of fit notes provision, with an IRR of 1.11 (95% CI 1.09 to 1.12) for every point increase in the Cambridge Multimorbidity Score. Prior consultations were associated with a slightly increased incidence rate of fit notes (IRR 1.02, 95% CI 1.02 to 1.02). However, prior consultations and comorbidity burden were highly correlated.

Finally, there was also geographic variation in the adjusted fit note incidence rates, with significantly higher rates seen in areas outside London, and individuals in the Northeast and Northwest having the highest rates (IRR 1.35, 95% CI 1.33 to 1.36; IRR 1.34, 95% CI 1.33 to 1.35, respectively).

Similar results were observed in the late-pandemic period (see Fig. 1 and Additional file 11). Estimated marginal effects from the regression models are presented in Additional files 10 and 12. As in Additional files 13 and 14, results from the sensitivity analysis were broadly consistent with the main results.

### Estimated cost of sickness absence

The weighted average duration of fit notes from NHS Digital data varied across the study years, with an overall increase observed in 2022 compared to the pre-pandemic period. In 2022, the average duration of fit notes was 29.8 days, marking an increase from 26.7 days in 2019 and 26.2 days in 2018.

**Fig. 2:**
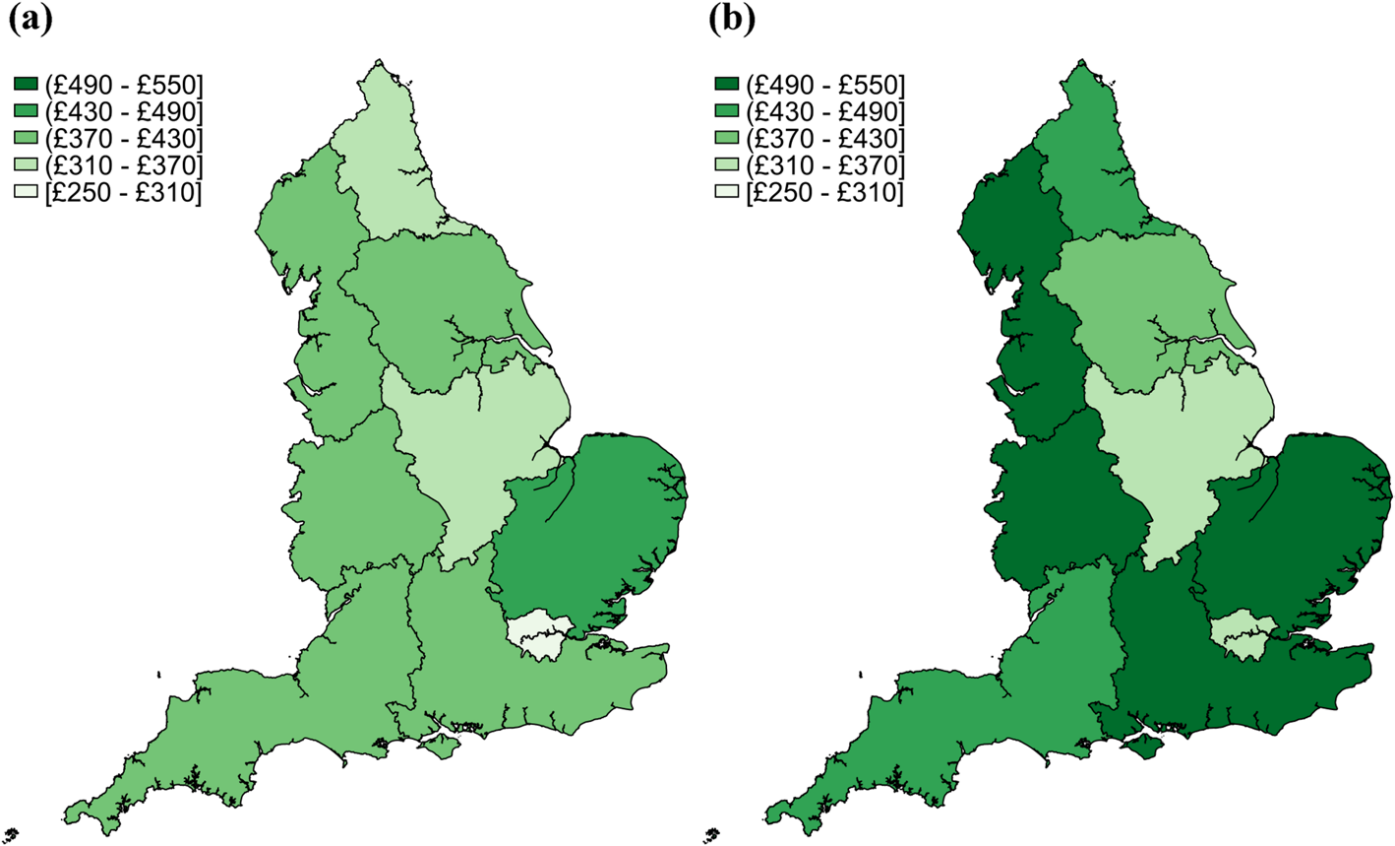
Individual average cost of sickness absence in each region in 2017–2019 and in 2022 in England. **a** Average cost of sickness absence in 2017–2019. **b** Average cost of sickness absence in 2022

Figure 2 and Additional file 15 show that the estimated individual average cost of sickness absence varied across regions in England from 2017 to 2022. The individual average annual cost of sickness absence, estimated based on the whole of England, rose from £378 in 2017 to £451 in 2022. In 2017, the highest costs were observed in the East of England (£457, 95% CI £449 to £465) and the Southwest (£429, 95% CI £424 to £433), while London had the lowest average cost (£259, 95% CI £257 to £261). A similar pattern was also observed in 2018, 2019 and 2022. As compared to the pre-pandemic period, there was a sharp increase in the costs of sickness absence across all regions in England in 2022. The East of England (£541, 95% CI £530 to £552), West Midlands (£511, 95% CI £506 to £516), and Northwest (£508, 95% CI £504 to £513) had the highest recorded costs, while London, despite an increase, remained the lowest at £318 (95% CI £315 to £321).

**Fig. 3:**
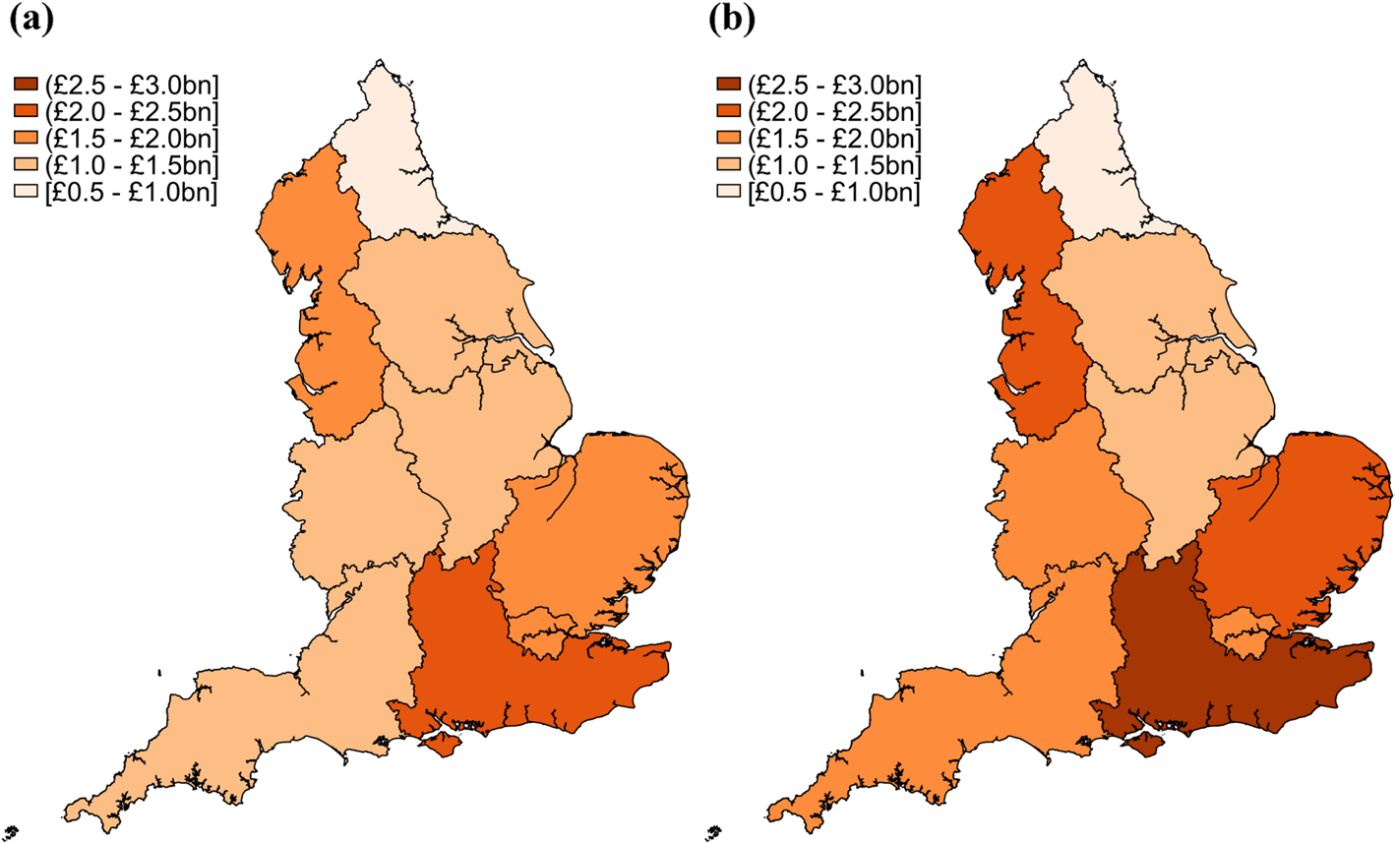
Regional total cost of sickness absence in 2017–2019 and in 2022 in England. **a** Total cost of sickness absence in 2017–2019. **b** Total cost of sickness absence in 2022

The estimated total costs of sickness absences are displayed in Fig. 3 and Additional file 16. The total estimated costs of sickness absence in England varied across regions between 2017 and 2022, with an overall upward trend. In 2017, the total cost for England was £13.3 billion (95% CI £13.3 to £13.4 billion), with the Southeast (£2.40 billion, 95% CI £2.38 to £2.42 billion) and Northwest (£1.85 billion, 95% CI £1.84 to £1.87 billion) incurring the highest regional costs. London also had a significant economic burden of £1.55 billion (95% CI £1.53 to £1.56 billion), despite having the lowest average individual sickness absence costs. By 2022, there was a substantial increase in the costs of sickness absence across all regions. The total cost for England was £15.9 billion (95% CI £15.8 to £15.9 billion), marking a sharp rise from previous years before the COVID-19 pandemic. The Southeast (£2.86 billion, 95% CI £2.83 to £2.88 billion) and Northwest remained (£2.34 billion, 95% CI £2.32 to £2.36 billion) the most affected regions, while London’s costs rose significantly to £1.90 billion (95% CI £1.88 to £1.92 billion).

### Medical reasons for fit note provision

Table 2 shows medical reasons for fit note provision. These sickness absence-related conditions reflect the ten leading causes of overall disease burden in England in 2022, as identified by the Global Burden of Disease (GBD) framework, for individuals aged 15–49 and 50–69 years, respectively. We were only able to attribute a medical diagnosis to around 30% of fit notes in our study period. Of these, depression and anxiety were the most prevalent conditions associated with sick note provision, followed by back pain, asthma and type 2 diabetes, both before and during the latter part of the COVID-19 pandemic. Less common reasons included osteoarthritis of the knee, falls, bipolar disorder, and hearing impairment. The proportion of fit notes attributed to each of these diagnoses remained broadly similar in the pre-pandemic and late-pandemic periods.

**Table 2:** Medical reasons for fit notes issued

Sickness reason	Jan 2017- Dec 2019		Mar 2022- Feb 2023	
	Number	Percentage (%)	Number	Percentage (%)
Depression	775,412	11.20	252,487	11.38
Anxiety	585,590	8.46	214,428	9.67
Back pain	501,608	7.24	132,628	5.98
Asthma	91,496	1.32	25,360	1.14
Type 2 diabetes	63,560	0.92	23,533	1.06
Neck pain	47,573	0.69	10,803	0.49
COPD	42,198	0.61	13,378	0.60
Migraine	35,071	0.51	10,978	0.49
Alcohol misuse	32,085	0.46	64,95	0.29
Osteoarthritis knee	22,741	0.33	10,555	0.48
Falls	22,284	0.32	6,404	0.29
Bipolar disorder	13,205	0.19	3,329	0.15
Hearing loss	8,675	0.12	2,961	0.13

Note: Each fit note was assigned a diagnostic category based on the most recent SNOMED-CT code recorded within one month before the fit note date. The table above shows the proportion of fit notes issued associated with each medical diagnosis relative to the total number of fit notes issued. The sickness absence-related conditions included represent the ten leading contributors to overall disease burden in 2022, according to the GBD framework, for individuals aged 15–49 and 50–69 years in England

*COPD *chronic obstructive pulmonary disease

## Discussion

### Findings

This study used primary care data to examine the frequency and economic impact of fit notes issued in primary care to the working-age population in England. Our findings showed that the age-standardised fit note rate increased from 23.5 per 100 person-years before the pandemic to 24.0 per 100 person-years in the late pandemic period. Fit note issuance was higher among females, older adults, Black ethnic groups, lower-income groups, people with obesity, current smokers, and those with comorbidities. Among the leading conditions considered in our study, mental health conditions, such as depression and anxiety, were the leading reasons for fit note issuance.

The economic burden of sickness absence increased significantly following the COVID-19 pandemic. Before the pandemic, the average annual cost of sickness absence per patient was £378 (95% CI £377 to £380), rising to £451 (95% CI £449 to £453) in the late pandemic period. At the national level, the total annual cost of sickness absence in England increased from £13.3 billion (95% CI £13.3 to £13.4 billion) pre-pandemic to £15.9 billion (95% CI £15.8 to £15.9 billion) during the latter part of the pandemic.

### Comparison with previous studies

To the best of our knowledge, this study is the first to estimate sickness absence and associated costs among the English working age population using health record data. While previous studies on sickness absences have been based on non-UK populations, healthcare workers, or relatively small sample sizes, this study included a large representative English working age population, covering the pre-COVID-19 pandemic period (2017–2019) and the latter part of the pandemic (2022–2023). By providing a comprehensive analysis of sickness absence across various demographic groups and contemporary periods, this study contributes valuable insights to the existing body of research while expanding the evidence base specific to the English population.

Our findings align with existing evidence that identifies higher sickness absence rates among females than males [35]. Additionally, a cohort study based in the UK (*n* = 4,580), France (*n* = 10,686), and Finland (*n* = 59,030) [36] found that lifestyle risk factors such as obesity, smoking, and heavy drinking were associated with increased sickness absence, which is consistent with our findings, although we did not examine the impact of alcohol use in this study. Similarly, other risk factors such as older age and mental health disorders have previously been linked to higher sickness absence rates [37].

Consistent with Dorrington et al. [38], we observed significant ethnic disparities, with individuals from Black ethnic groups receiving more fit notes than their White counterparts, while those from Asian and Mixed ethnic groups received fewer fit notes. Similarly, our results align with existing evidence showing higher fit note rates among individuals living in more socioeconomically deprived areas [38]. Furthermore, regional disparities in sickness absence rates (i.e., the percentage of working hours lost due to sickness or injury) have been documented in previous literature. For example, according to the Office for National Statistics, where sickness absence was measured using data from the Labour Force Survey, London had lower sickness absence rates compared to other parts of the UK, which corresponds with our findings of lower fit note rates in this region [39].

This study provides a health record-based estimate of the costs of sickness absence, showing an increase from £13.3 billion in 2017 to £15.9 billion in the late pandemic period, with per-person costs rising from £378 to £451 annually. This study also highlights mental health conditions (depression and anxiety) as the leading causes of sickness absence, aligning with previous studies [40, 41]. Prior research [40, 41] used national survey data and employer-reported statistics to estimate broader economic losses, including lost productivity and informal caregiving. This study focused specifically on sickness absence costs estimated from primary care electronic health records, which allows for a clinically validated and detailed analysis of health-related absence.

Black & Forst [42] estimated the sickness absence cost to the UK of nearly £9 billion in 2010, and a 2020 analysis found it accounted for 0.57% of GDP, which is approximately £15.7 billion [43]. Unlike this study, these studies relied on workplace surveys and associated economic modelling rather than individual health records. The concordance of our findings with existing economic literature demonstrates that primary healthcare record data is likely to be a valid data source for modelling economic losses related to sickness absence. By providing late-pandemic cost estimates at both individual and national levels, this study builds on the existing evidence to better understand the economic impact and variation of sickness absence in the English working-age population.

Our study demonstrates that while the rate of fit note provision increased over the study period, the risk factors during both pre-pandemic and late-pandemic periods were similar. The increase in fit note provision could potentially be due to a shift in the characteristics of the population, such as increasing levels of income deprivation and obesity, or due to risk factors and conditions that were not included in our analysis, such as social isolation and long COVID [44].

In addition, changes in employment may also have influenced the observed trends in fit note use. Excluding the acute COVID-19 period, the overall employment rate in the UK remained broadly stable in 2017–2019 and 2022–2023 [45]. Trends in wages and unfilled vacancies also showed little change over the study window [45]. However, there was widespread adoption of remote and hybrid working arrangements during and after the COVID-19 pandemic. Remote work may have reduced the need for formal sickness certification among individuals with milder symptoms, who could continue working from home or rely on self-certification [46], although the extent of this behavioural adaptation likely varies by factors such as job type (e.g., manual versus office-based work). A formal medical certificate was therefore potentially more likely to be requested for more severe illness or longer-term sick leave [46]. Therefore, the observed increase in the average duration of fit notes could partly reflect a shift towards more severe cases rather than uniformly longer episodes. Our cost estimates capture certified sickness absence only and do not account for episodes managed through remote work or without formal certification. As a result, they may underestimate the overall burden of sickness absence in England.

### Strengths and limitations

We examined sickness absence and associated costs using large, nationally representative healthcare record data from primary care in England. Unlike previous studies that focus solely on sickness absence rates, this study estimated the economic loss due to sickness absence in England, providing policymakers with quantifiable evidence of the financial burden associated with sickness absences before and during the latter part of the COVID-19 pandemic, and variation by demography and clinical risk factors. The large sample size and representativeness of the CPRD Aurum database to the English population also make our findings generalisable to the working age population in England. This study also demonstrates the feasibility of using fit note data from primary care records to estimate the cost of sickness absence at a population level.

However, this study is subject to several limitations. First, the analysis relied on routinely collected health record data, which restricts the available information to what was routinely recorded in clinical practice. There is additional information that would have been useful for our analyses, such as employment status, educational level, or access to state benefits, which were not available in our datasets. It was therefore impossible to specify whether an individual was employed, unemployed, retired or in full-time education, and whether fit notes were issued for an individual in work or who was out of work but aimed to apply for social benefits. Our estimates were therefore made on the assumption that the individuals receiving fit notes would otherwise be in paid work.

Furthermore, since clinical diagnoses are not routinely coded alongside fit notes in primary care, we identified the medical reason for each fit note using the SNOMED-CT diagnosis recorded within one month prior to and closest to the fit note issuance date, as a proxy for the certified condition. However, it is possible that a health condition recorded closest to the date when a fit note was issued might not necessarily be the reason for sickness indicated on the fit note, which is often entered in free text rather than entered as coded data. Moreover, only around 30% of fit notes could be assigned a diagnosis using this method, due to the absence of a qualifying coded entry within the specified time window. This level of missing diagnostic data might not be random, potentially reflecting systematic differences in factors such as coding practices, clinical complexity, or patient characteristics [47]. As such, findings on the distribution of diagnostic reasons for sickness absence should be interpreted with caution. They reflect the subset of fit notes with available coded diagnoses, which may not fully represent the underlying distribution across all certified absences. More complete data sources would help to characterise these patterns more precisely and representatively in future research.

Finally, as CPRD Aurum lacked data on the duration of individual fit notes, we used population-level average fit note durations from NHS Digital data to inform our cost estimates. However, these population-level averages may not accurately reflect variation in sickness absence duration within our study cohorts. Thus, we were unable to examine risk factors for prolonged sickness absences, which is an important dimension of work incapacity. This suggests a key area for future research, particularly where individual-level data on the duration of fit notes are available.

### Implications

This study has several implications for policymaking and future research. First, our findings confirm the substantial and rising economic costs of sickness absence in England, which increased from £13.3 billion before the pandemic to £15.9 billion during the latter part of the pandemic. Given that the total Universal Credit bill was £73.2 billion in 2022/23, the £2.4 billion increase in sickness absence costs is equivalent to around one quarter of the rise in the welfare bill over the same period (from £63.1 billion to £73.2 billion) [48]. These findings raise public awareness of the need for sustained effort to tackle sickness-related economic inactivity.

Investment is needed in healthcare services and workplaces to support patients and employees to remain in work without it being detrimental to their health. Likewise, active labour market policies that support individuals and firms in continuing the employment of those with health conditions may bring inactivity rates closer to those in other G7 countries. Future research might usefully evaluate the cost-effectiveness of different interventions, including those at the primary care level, to understand which strategies are most effective and sustainable in reducing sickness absence rates and supporting long-term job retention. Notably, costs in London were lower than in other regions, in both aggregate and per capita terms, despite higher wages. This likely reflects the concentration of fit note issuance in communities with the highest rates of deprivation, amongst which there is less cross-regional variation in incomes.

The study also identified disparities in fit note issuance, with higher fit note rates being observed among specific sociodemographic groups, such as females, older adults, those from Black ethnic groups, and those with higher income deprivation, as well as those with obesity, smokers and patients with a greater number of comorbidities. These findings suggest the need for more targeted support to different demographic populations to mitigate these inequalities and highlight the important links between health and economic activity. This also highlights the need for further research to understand the underlying causes of these demographic disparities. This includes a need to understand the gender differences observed, including the potential contribution of pregnancy and menopausal symptoms on sickness absence, which we did not account for in this study. Moreover, this study identifies that mental health conditions, such as depression and anxiety, are among the leading causes of sickness absence. This suggests that mental health support in the working-age population may be particularly important for preventing sickness absence and associated economic losses.

In England, doctors play a key role in determining a patient’s ability to return to work and in shaping patients’ and employers’ perceptions of that ability when issuing fit notes. However, GPs have reported a lack of confidence in issuing fit notes effectively [49]. Furthermore, since 2022, other healthcare professionals, including nurses, occupational therapists, pharmacists, and physiotherapists, have also been authorised to certify fit notes to patients. As such, clear guidance and training for healthcare professionals on how to assess work capability and complete fit notes in a way that benefits both patients and employers, while maintaining doctor-patient relationships, are needed. This will ensure fit notes serve as an effective tool for managing sickness absence and workplace participation.

## Conclusions

Sickness absence rates among working-age adults in England have risen in recent years, representing an estimated £16 billion in annual employment costs in the latter part of the pandemic. The rates of sickness absence vary substantially by demography, health status, income and geography, and poor mental health is likely to be a key driver of the upward trend. These findings highlight the importance of investing in public health to boost economic activity, as well as workplace and health service interventions to help people remain in work while supporting their health. Even a small decline in sickness absence rates could bring about significant economic returns.

## Supplementary Information

Additional file 1: Additional supporting files.

## Data Availability

Data are not publicly available. Access to Clinical Practice Research Datalink (CPRD) data is subject to a license agreement and a protocol approval process overseen by CPRD’s research data governance. A guide to data access is provided on the CPRD website (https:/www.cprd.com/access-data/organisation-approval). This study was approved by the CPRD Independent Scientific Advisory Committee (Study reference ID: 23\_003538).
