# Supplementary material for: Sickness absence in the working age population: a retrospective cohort study using primary care health record data
Source: BMC Public Health. 2026 Feb 5;26:829. doi: 10.1186/s12889-026-26296-6 (PMC12973743; doi:10.1186/s12889-026-26296-6)
Supplement: Supplementary file 1 — Additional file 1: Additional supporting files. [file 12889_2026_26296_MOESM1_ESM.docx]

**Additional supporting material**

**Sickness absence in the working age population: a retrospective cohort study using primary care health record data**

**Table of Contents**

[Additional file 1 Flow chart of study population for pre-pandemic cohort (2017-2019) 3](#_Toc216252918)

[Additional file 2 Flow chart of study population for late-pandemic cohort (2022-2023) 4](#_Toc216252919)

[Additional file 3 Cost estimation and key assumptions 5](#_Toc216252920)

[Additional file 4 Total number of fit notes issued across different subgroups in 2017 8](#_Toc216252921)

[Additional file 5 Total number of fit notes issued in different subgroups in 2022 9](#_Toc216252922)

[Additional file 6 Age-standardised fit note rates 10](#_Toc216252923)

[Additional file 7 Table of explanatory variables included in the study 13](#_Toc216252924)

[Additional file 8 Comorbidity included for comorbidity score 14](#_Toc216252925)

[Additional file 9 Risk factors and fit notes: IRR from random-effect negative binomial regressions based on the pre COVID-19 pandemic cohort (2017-2019) 15](#_Toc216252926)

[Additional file 10 Risk factors and fit notes: Marginal effects from negative binomial regressions based on the pre COVID-19 pandemic cohort (2017-2019) 18](#_Toc216252927)

[Additional file 11 Risk factors and fit notes: IRR from negative binomial regressions based on the late COVID-19 pandemic cohort (2022) 20](#_Toc216252928)

[Additional file 12 Risk factors and fit notes: Marginal effects from negative binomial regressions based on the late COVID-19 pandemic cohort (2022) 22](#_Toc216252929)

[Additional file 13 Risk factors and fit notes: IRR from fixed-effect Poisson regressions based on the pre-pandemic cohort (2017-2019) 24](#_Toc216252930)

[Additional file 14 Risk factors and fit notes: IRR from fixed-effect Poisson regressions based on the late COVID-19 pandemic cohort (2022) 26](#_Toc216252931)

[Additional file 15 Average cost estimation for English nations in each cohort 28](#_Toc216252932)

[Additional file 16 Estimated regional total cost of sickness absence 30](#_Toc216252933)

[Additional file 17 Sensitivity analysis: Average cost estimation for English nations in each cohort 32](#_Toc216252934)

[Additional file 18 Sensitivity analysis: Regional total cost of sickness absence 33](#_Toc216252935)

[Additional file 19 Medical code lists used for fit note (“not fit for work”) 35](#_Toc216252936)

[Additional file 20 Patient and public involvement (PPIE) 36](#_Toc216252937)

## Additional file 1 Flow chart of study population for pre-pandemic cohort (2017-2019)

Total patients recorded in pre-pandemic (2017-2019) cohort aged between 18-65

N=11,406,264

Total sample in cohort 2017-2019: who do not have a recorded fit note between Jan 2017-Dec 2019:

N=9,559,248

N

Total sample in cohort 2017-2019: who have at least one recorded fit note between Jan 2017-Dec 2019:

N=1,847,016

N

Total patients recorded in pre-pandemic cohort included in regression analysis

N=9,341,818

Total patients excluded due to:

No data were matched to IMD income data

N=2,064,446

## Additional file 2 Flow chart of study population for late-pandemic cohort (2022-2023)

Total patients recorded in late-pandemic (2022-2023) cohort aged between 18-65

N=10,044,331

Total sample in cohort 2022: who do not have a recorded fit note between March 2022-Feb 2023:

N=9,200,576

N

Total sample in cohort 2022: who have at least one recorded fit note between March 2022-Feb 2023:

N=843,775

N

Total patients recorded in late-pandemic (2022-2023) cohort included in regression analysis

N=7,520,270

Total patients excluded due to:

No data were matched to IMD income data

N=2,524,061

## Additional file 3 Cost estimation and key assumptions

The economic cost of sickness absence for England’s population was estimated combining estimates of the unit economic cost of sickness absence (i.e., average individual income per day) and the length of sickness absence (i.e., the number of days off work), using multiple data sources.

Specifically, in the absence of earnings information for individual patients they are attributed with the average earnings of individuals in the same region, living in communities with the same Index of Multiple Deprivation score. These income data were obtained from the Office for National Statistics (ONS) Admin-based income statistics for England and Wales (tax year ending 2018) [1] in the main analysis, which provided detailed annual earnings estimates stratified by geographic region and Index of Multiple Deprivation (IMD) levels. In sensitivity analysis, income data were obtained from ONS income statistics from Pay As You Earn (PAYE) and benefits for tax year ending 2016 [2], which provided earnings estimates stratified by lower layer Super Output Area (LSOA) and income deciles. These earnings data were subsequently linked to individual records within the CPRD Aurum database based on matching IMD and regional identifiers. Given that these earnings estimates pertained specifically to the 2018 tax year, data for the years 2017, 2019, and 2022 were adjusted to reflect changes in average earnings over time [2]. Adjustments were based on annual average earnings growth rates, calculated from the ONS published statistics on UK average weekly earnings growth spanning from 2001 to 2024. These earnings estimates were then transformed into the unit cost of workplace absence for workers, on the assumption that the economic loss was equal to earnings, by dividing annual earnings by 260 (52 weeks * 5 working days per week). This is a conservative assumption as it ignores annual leave. Denote the set of region and IMD combinations as $K$with typical element $k$ then the unit cost in community k is given as

$$Unit Cost_{k}=\frac{{Average Earnings}_{k}}{260}$$

To calculate the length of sickness absences associated with fit notes data were extracted from NHS Digital’s reports on fit notes issued by GP practices in England, covering the period from Jan 2017- Dec 2017, Jan 2018 – Dec 2018, Jan 2019 – Dec 2019 and Mar 2022 – Feb 2023 [3]. The data provided counts of fit notes by diagnosis (ICD-10 chapter of condition) and by duration, categorised into intervals spanning days (1–7, 8–14, 15–21, 22–28) and weeks (5–12, 13–20, >20) [3]. These detailed data were used to estimate the distribution for England of workdays lost due to sickness absence in different years, for each health condition. The average of this distribution for each ICD-10 chapter was computed as the midpoint of each category multiplied by the proportion of fit notes falling in that duration category. For example, a midpoint of 4 days was used for the 1-7 day category, 11 days for 8-14 days, 18 days for 15-21 days, and so on. This produced an estimated average duration of about 30 days per fit note for those lasting less than 20 weeks. Fit notes classified as indefinite (Indefinite), were excluded due to the absence of a defined upper bound. 5 additional working days were added to the average duration of fit note when estimating the cost of sickness absence to account for absences during the self-certification period. Mathematically, if

$$Average days lost per fit note= \sum_{d\in D} \frac{\left( Max_{d}-Min_{d} \right)}{2}\times p_{dj}$$

Where $p_{dj}$ is the proportion of fit notes in the interval *d* for diagnosis $j$.

Estimates of the cost of workplace absence were then obtained by combining these two sets of estimates. The cost of workplace absence for an individual with diagnosis $j$ who lives in a community of type (deprivation and region) $k$ is:

$$Average Cost of absence_{jk}=Average days lost per fit note_{j}\times Unit Cost_{k}$$

Denoting the number of people with diagnosis $j$ living in a community of type k as $N_{jk}$, the total cost is:

$$Total output loss= \sum_{k\in K} \sum_{j\in J} Average Cost of absence_{jk}N_{jk}$$

## Additional file 4 Total number of fit notes issued across different subgroups in 2017

| **Variable** | **Total number of fit notes** | **Kruskal-Wallis non-parametric test** |
| --- | --- | --- |
| **Sex** |  |  |
| Females | 1,142,649 | P<0.001 |
| Males | 875,918 |  |
| **Age group (age in 2017)** |  |  |
| 18-30 | 372,808 | P<0.001 |
| 31-40 | 409,893 |  |
| 41-50 | 496,930 |  |
| 51-60 | 567,532 |  |
| 61-65 | 171,404 |  |
| **Ethnicity** |  |  |
| White | 1,568,085 | P<0.001 |
| Asian | 125,908 |  |
| Black | 89,514 |  |
| Other | 10,562 |  |
| Mixed | 30,525 |  |
| **BMI category** |  |  |
| Underweight | 52,492 | P<0.001 |
| Normal | 578,146 |  |
| Overweight | 542,631 |  |
| Obese | 580,357 |  |
| **Smoking status** |  |  |
| Never smoked | 614,892 | P<0.001 |
| Ex-smoker | 566,479 |  |
| Current smoker | 803,707 |  |
| **Region** |  |  |
| East Midlands | 45,859 | P<0.001 |
| East of England | 60,986 |  |
| London | 290,797 |  |
| Northeast | 106,583 |  |
| Northwest | 477,657 |  |
| Southeast | 355,228 |  |
| Southwest | 219,014 |  |
| West Midlands | 374,760 |  |
| Yorkshire and the Humber | 75,587 |  |

*Notes:* For subgroups with missing data, the total number of fit notes is not reported.

## Additional file 5 Total number of fit notes issued in different subgroups in 2022

| **Variable** | **Total number of fit notes** | **Kruskal-Wallis non-parametric test** |
| --- | --- | --- |
| **Sex** |  |  |
| Females | 1,210,772 | P<0.001 |
| Males | 859,445 |  |
| **Age group** |  |  |
| 18-30 | 356,768 | P<0.001 |
| 31-40 | 443,635 |  |
| 41-50 | 458,769 |  |
| 51-60 | 575,298 |  |
| 61-65 | 232,747 |  |
| **Ethnicity** |  |  |
| White | 1,592,236 | P<0.001 |
| Asian | 182,596 |  |
| Black | 112,808 |  |
| Other | 18,944 |  |
| Mixed | 42,374 |  |
| **BMI category** |  |  |
| Underweight | 57,128 | P<0.001 |
| Normal | 558,855 |  |
| Overweight | 560,160 |  |
| Obese | 659,140 |  |
| **Smoking status** |  |  |
| Never smoked | 610,495 | P<0.001 |
| Ex-smoker | 679,902 |  |
| Current smoker | 712,509 |  |
| **Region** |  |  |
| East Midlands | 36,046 | P<0.001 |
| East of England | 54,177 |  |
| London | 337,473 |  |
| Northeast | 108,059 |  |
| Northwest | 510,954 |  |
| Southeast | 350,881 |  |
| Southwest | 195,243 |  |
| West Midlands | 420,189 |  |
| Yorkshire and the Humber | 57,195 |  |

*Notes:* For subgroups with missing data, the total number of fit notes is not reported.

## Additional file 6 Age-standardised fit note rates

Additional file 6 (1) Age-standardised fit note rates (per 100-person year) in 2017 and 2018

| **Subgroups** | **2017: Crude rate** | **2017: Age-standardised rate** | **95% CIs** | **2018: Crude rate** | **2018: Age-standardised rate** | **95% CIs** |
| --- | --- | --- | --- | --- | --- | --- |
| **Whole population** | **23.57** | **23.57** | **23.54, 23.60** | **23.37** | **23.38** | **23.35, 23.42** |
| **Sex** |  |  |  |  |  |  |
| Male | 19.82 | 19.85 | 19.81, 19.88 | 19.41 | 19.45 | 19.41, 19.48 |
| Female | 27.57 | 27.56 | 27.51, 27.60 | 27.6 | 27.6 | 27.56, 27.65 |
| **Ethnicity** |  |  |  |  |  |  |
| Asian | 16.34 | 17.68 | 17.59, 17.77 | 16.1 | 17.43 | 17.34, 17.52 |
| Black | 26.05 | 26.28 | 26.13, 26.43 | 25.82 | 26.01 | 25.87, 26.15 |
| Missing | 16.47 | 17.13 | 17.05, 17.20 | 15.8 | 16.47 | 16.41, 16.55 |
| Mixed | 12.75 | 14.12 | 13.85, 14.38 | 12.13 | 13.61 | 13.36, 13.86 |
| Other | 23.97 | 25.23 | 24.97, 25.48 | 23.19 | 24.37 | 24.12, 24.61 |
| White | 25.87 | 25.57 | 25.54, 25.60 | 25.77 | 25.45 | 25.42, 25.49 |
| **BMI category** |  |  |  |  |  |  |
| Underweight (<18) | 22.64 | 28.16 | 27.92, 28.41 | 21.19 | 26.47 | 26.24, 26.71 |
| Normal (18-24) | 20.43 | 20.81 | 20.76, 20.86 | 20.16 | 20.55 | 20.50, 20.60 |
| Overweight (25-29) | 24.46 | 23.92 | 23.86, 23.98 | 24.49 | 23.9 | 23.84, 23.96 |
| Obese (>=30) | 35.3 | 34.83 | 34.75, 34.91 | 35.48 | 34.99 | 34.91, 35.07 |
| Missing | 16.16 | 17.12 | 17.05, 17.18 | 16.16 | 17.32 | 17.25, 17.38 |
| **Region** |  |  |  |  |  |  |
| East Midlands | 21.57 | 22.21 | 22.03, 22.39 | 21.21 | 21.96 | 21.79, 22.14 |
| East of England | 18.35 | 18.15 | 18.02, 18.28 | 18.18 | 17.95 | 17.81, 18.08 |
| London | 17.32 | 18.07 | 18.01, 18.13 | 17.06 | 17.83 | 17.77, 17.89 |
| Northeast | 32.9 | 33.15 | 32.99, 33.31 | 32.42 | 32.77 | 32.61, 32.93 |
| Northwest | 29.92 | 29.85 | 29.78, 29.92 | 29.63 | 29.58 | 29.52, 29.66 |

| **Subgroups** | **2017: Crude rate** | **2017: Age-standardised rate** | **95% CI** | **2018: Crude rate** | **2018: Age-standardised rate** | **95% CI** |
| --- | --- | --- | --- | --- | --- | --- |
| Southeast | 19.07 | 18.96 | 18.90, 19.01 | 19.14 | 19.08 | 18.98, 19.10 |
| Southwest | 25.38 | 25.3 | 25.23, 25.37 | 25.82 | 25.73 | 25.62, 25.84 |
| West Midlands | 27.51 | 27.47 | 27.39, 27.54 | 27.12 | 27.07 | 27.00, 27.17 |
| Yorkshire and the Humber | 25.51 | 25.83 | 25.68, 25.99 | 24.64 | 25.08 | 24.88, 25.19 |
| **Smoking status** |  |  |  |  |  |  |
| Never smoked | 17.13 | 17.47 | 17.43, 17.51 | 17.33 | 17.68 | 17.64, 17.72 |
| Ex-smoker | 25.77 | 25.12 | 25.10, 25.18 | 25.86 | 25.22 | 25.16, 25.28 |
| Current smoker | 33.63 | 33.61 | 33.55, 33.67 | 32.96 | 32.92 | 32.86, 33.00 |
| Missing | 8.66 | 8.4 | 8.29, 8.50 | 9.33 | 9.1 | 8.99, 9.20 |

Additional file 6 (2) Age-standardised fit note rates (per 100-person year) in 2019 and 2022

| **Subgroups** | **2019: Crude rate** | **2019: Age-standardised rate** | **95% CIs** | **2022: Crude rate** | **2022: Age-standardised rate** | **95% CIs** |
| --- | --- | --- | --- | --- | --- | --- |
| **Whole population** | **23.57** | **23.57** | **23.54, 23.60** | **23.86** | **24.03** | **24.00, 24.06** |
| **Sex** |  |  |  |  |  |  |
| Male | 19.45 | 19.48 | 19.44, 19.51 | 19.04 | 19.28 | 19.24, 19.31 |
| Female | 27.99 | 27.98 | 27.93, 28.02 | 29.1 | 29.19 | 29.15, 29.24 |
| **Ethnicity** |  |  |  |  |  |  |
| Asian | 16.41 | 17.77 | 17.68, 17.86 | 19.1 | 20.92 | 20.83, 21.00 |
| Black | 26.08 | 26.19 | 26.05, 26.34 | 28.06 | 28.54 | 28.40, 28.68 |

| **Subgroups** | **2019: Crude rate** | **2019: Age-standardised rate** | **95% CIs** | **2022: Crude rate** | **2022: Age-standardised rate** | | **95% CIs** |
| --- | --- | --- | --- | --- | --- | --- | --- |
| Missing | 15.36 | 15.98 | 15.91, 16.05 | 13.13 | | 13.96 | 13.88, 14.03 |
| Mixed | 12.31 | 13.93 | 13.66, 14.14 | 15.05 | | 17.09 | 17.66, 18.15 |
| Other | 23.5 | 24.79 | 24.56, 25.03 | 24.86 | | 26.91 | 26.66, 27.14 |
| White | 26.02 | 25.7 | 25.66, 25.73 | 26.11 | | 25.79 | 25.76, 25.83 |
| **BMI category** |  |  |  |  | |  |  |
| Underweight (<18) | 20.93 | 25.86 | 25.62, 26.09 | 20.94 | | 26.83 | 26.59, 27.06 |
| Normal (18-24) | 20.19 | 20.54 | 20.49, 20.58 | 19.84 | | 20.38 | 20.33, 20.43 |
| Overweight (25-29) | 24.83 | 24.21 | 24.14, 24.27 | 25.26 | | 24.53 | 24.47, 24.59 |
| Obese (>=30) | 36.28 | 35.82 | 35.74, 35.96 | 37.97 | | 37.16 | 37.08, 37.23 |
| Missing | 16.42 | 17.51 | 17.44, 17.57 | 14.39 | | 16.19 | 16.12, 16.26 |
| **Region** |  |  |  |  | |  |  |
| East Midlands | 20.31 | 21.19 | 21.01, 21.36 | 21.52 | | 23.3 | 23.09, 23.51 |
| East of England | 17.73 | 17.54 | 17.41, 17.68 | 17.32 | | 17.14 | 17.04, 17.27 |
| London | 16.93 | 17.63 | 17.58, 17.69 | 17.53 | | 18.56 | 18.50, 18.61 |
| Northeast | 33.87 | 34.12 | 33.95, 34.28 | 35.02 | | 35.38 | 35.21, 35.55 |
| Northwest | 30.47 | 30.39 | 30.32, 30.46 | 30.99 | | 31.01 | 30.95, 31.08 |
| Southeast | 19.22 | 19.11 | 19.05, 19.16 | 18.75 | | 18.67 | 18.62, 18.73 |
| Southwest | 25.5 | 25.43 | 25.33, 25.54 | 24.22 | | 24.19 | 24.10, 24.29 |
| West Midlands | 27.44 | 27.37 | 27.29, 27.44 | 29.68 | | 29.67 | 29.60, 29.74 |
| Yorkshire and the Humber | 25.7 | 26.19 | 26.03, 26.36 | 26.11 | | 27.35 | 27.16, 27.54 |
| **Smoking status** |  |  |  |  | |  |  |
| Never smoked | 17.71 | 18.02 | 17.98, 18.06 | 17.85 | | 18.37 | 18.33, 18.41 |
| Ex-smoker | 26.29 | 25.66 | 25.60, 25.73 | 20.87 | | 27.22 | 27.16, 27.28 |
| Current smoker | 33.09 | 33 | 32.93, 33.06 | 32.75 | | 32.86 | 32.80, 32.93 |
| Missing | 10.15 | 9.79 | 9.69, 9.89 | 10.21 | | 10.96 | 10.85, 11.08 |

## Additional file 7 Table of explanatory variables included in the study

| **Variable** | **Description** | **Specification** |
| --- | --- | --- |
| Age | Age in years at index date (cohort entry). | Categorical variable: 18-30, 31-40, 41-50, 51-60, 61-65 |
| Sex | Biological sex recorded in the patient record. | Binary variable: equal 1 if male; otherwise, if female |
| Body mass index (BMI) category | BMI calculated as weight (kg) divided by height squared (m²). Classified according to WHO thresholds. | Categorical variable: Underweight (<18.5 kg/m2), Normal weight (18.5 – 24.9 kg/m2), Overweight (25 – 29.9 kg/m, Obese (≥30 kg/m2), Missing |
| Index of Multiple Deprivation (IMD) income domain deciles | Measure of socioeconomic deprivation based on income domain. | Categorial variable: IMD income deciles range from 1 (least deprived) to  10 (most deprived) |
| Smoking status | Self-reported smoking behaviour recorded in primary care records. | Categorial variable: Never smoked, Ex-smoker, Current smoker, Missing |
| Ethnicity | Self-identified ethnicity as recorded in the patient’s health record. | Categorical variable: White, Black, Mixed, Asian, Other, Missing |
| Practice ID | Identifier for the general practice where the patient is registered. Used to account for clustering. | Categorial variable (unique practice identifier) |
| Number of Consultations in the past 12 months | Total number of consultations (face-to-face, telephone, remote, etc.) with healthcare professionals in the 12 months prior to index date. | Continuous |
| Comorbidity score | Comorbidity burden based on presence of 21 chronic conditions. See Supplementary Table S12 for definitions and scoring. | Continuous |
| Region | Geographical region based on registered practice location. | Categorial variable: Northeast, Northwest, East Midlands, West Midlands, Southeast, Southwest, East of England, London, Yorkshire and The Humber |

## Additional file 8 Comorbidity included for comorbidity score

| **21 included conditions** | **Variable** | **Weight** |
| --- | --- | --- |
| **1** | Alcohol problems | 0.792243 |
| **2** | Anxiety or depression | 0.324207 |
| **3** | Atrial fibrillation | 0.334891 |
| **4** | Cancer in the last 5years | 1.202615 |
| **5** | Chronic kidney disease | 0.213652 |
| **6** | Chronic liver disease and viral hepatitis | 0.68621 |
| **7** | Constipation | 0.383006 |
| **8** | COPD | 0.702181 |
| **9** | Dementia | 0.938001 |
| **10** | Diabetes | 0.29467 |
| **11** | Disorder of prostate | -0.18781 |
| **12** | Epilepsy | 0.477465 |
| **13** | Heart failure | 0.505245 |
| **14** | Irritable bowel syndrome | -0.20368 |
| **15** | Learning disability | 0.637273 |
| **16** | Multiple sclerosis | 0.761606 |
| **17** | Painful condition | 0.445521 |
| **18** | Parkinsonism | 0.546194 |
| **19** | Peripheral vascular disease | 0.334558 |
| **20** | Psycho active substance misuse | 0.449321 |
| **21** | Schizophrenia or bipolar disorder | 0.482469 |

## Additional file 9 Risk factors and fit notes: IRR from random-effect negative binomial regressions based on the pre COVID-19 pandemic cohort (2017-2019)

|  | **Model 1** | | **Model 2** | | **Model 3** | |
| --- | --- | --- | --- | --- | --- | --- |
|  | **IRR** | **95% CI** | **IRR** | **95% CI** | **IRR** | **95% CI** |
| **Sex** |  |  |  |  |  |  |
| Female † | 1 | [1.00, 1.00] | 1 | [1.00, 1.00] | 1 | [1.00, 1.00] |
| Male | 0.71*** | [0.70, 0.71] | 0.70*** | [0.70, 0.71] | 0.72*** | [0.72, 0.72] |
| **Age** |  |  |  |  |  |  |
| 18-30 † | 1 | [1.00, 1.00] | 1 | [1.00, 1.00] | 1 | [1.00, 1.00] |
| 31-40 | 0.94*** | [0.94, 0.94] | 0.95*** | [0.94, 0.95] | 0.95*** | [0.94, 0.95] |
| 41-50 | 1.00 | [1.00, 1.01] | 1.03*** | [1.03, 1.04] | 1.02*** | [1.02, 1.03] |
| 51-60 | 1.08*** | [1.07, 1.08] | 1.11*** | [1.11, 1.12] | 1.09*** | [1.09, 1.10] |
| 61-65 | 0.94*** | [0.94, 0.95] | 0.99*** | [0.98, 0.99] | 0.95*** | [0.94, 0.96] |
| **Ethnicity** |  |  |  |  |  |  |
| White † | 1 | [1.00, 1.00] | 1 | [1.00, 1.00] | 1 | [1.00, 1.00] |
| Asian | 0.80*** | [0.80, 0.81] | 0.78*** | [0.77, 0.78] | 0.78*** | [0.77, 0.78] |
| Black | 1.25*** | [1.24, 1.25] | 1.18*** | [1.17, 1.19] | 1.19*** | [1.18, 1.20] |
| Missing | 0.80*** | [0.79, 0.80] | 0.80*** | [0.79, 0.80] | 0.81*** | [0.80, 0.81] |
| Mixed | 0.68*** | [0.67, 0.69] | 0.69*** | [0.67, 0.70] | 0.69*** | [0.68, 0.71] |
| Other | 1.06*** | [1.05, 1.08] | 1.04*** | [1.03, 1.05] | 1.05*** | [1.03, 1.06] |
| **BMI** |  |  |  |  |  |  |
| Normal (18.5-24.9) † | 1 | [1.00, 1.00] | 1 | [1.00, 1.00] | 1 | [1.00, 1.00] |
| Underweight (<18.5) | 1.11*** | [1.10, 1.12] | 1.09*** | [1.08, 1.10] | 1.09*** | [1.08, 1.10] |
| Overweight (25-29.9) | 1.16*** | [1.16, 1.17] | 1.16*** | [1.15, 1.16] | 1.15*** | [1.14, 1.15] |
| Obese (>=30) | 1.47*** | [1.46, 1.47] | 1.43*** | [1.42, 1.44] | 1.39*** | [1.39, 1.40] |
| Missing | 0.97*** | [0.96, 0.97] | 0.96*** | [0.96, 0.97] | 0.97*** | [0.97, 0.98] |
| **Region** |  |  |  |  |  |  |
| London † | 1 | [1.00, 1.00] | 1 | [1.00, 1.00] | 1 | [1.00, 1.00] |
| East Midlands | 1.19*** | [1.17, 1.21] | 1.29*** | [1.27, 1.31] | 1.28*** | [1.26, 1.30] |
| East of England | 1.01* | [1.00, 1.03] | 1.15*** | [1.13, 1.16] | 1.16*** | [1.15, 1.18] |
| Northeast | 1.38*** | [1.37, 1.40] | 1.35*** | [1.33, 1.36] | 1.35*** | [1.33, 1.36] |
| Northwest | 1.36*** | [1.35, 1.37] | 1.38*** | [1.37, 1.39] | 1.34*** | [1.33, 1.35] |
| Southeast | 1.08*** | [1.07, 1.09] | 1.22*** | [1.21, 1.23] | 1.21*** | [1.20, 1.22] |
| Southwest | 1.27*** | [1.26, 1.28] | 1.36*** | [1.35, 1.37] | 1.31*** | [1.30, 1.32] |
| West Midlands | 1.28*** | [1.27, 1.29] | 1.32*** | [1.31, 1.33] | 1.31*** | [1.30, 1.32] |
| Yorkshire and The Humber | 1.24*** | [1.22, 1.25] | 1.30*** | [1.29, 1.32] | 1.30*** | [1.28, 1.31] |
| **Smoking Status** |  |  |  |  |  |  |
| Never smoked † | 1 | [1.00, 1.00] | 1 | [1.00, 1.00] | 1 | [1.00, 1.00] |
| Ex-smoker | 1.28*** | [1.28, 1.29] | 1.27*** | [1.26, 1.27] | 1.25*** | [1.25, 1.26] |
| Current smoker | 1.61*** | [1.60, 1.61] | 1.53*** | [1.53, 1.54] | 1.52*** | [1.51, 1.52] |
| Missing | 0.76*** | [0.75, 0.76] | 0.76*** | [0.76, 0.77] | 0.77*** | [0.76, 0.77] |
| **Comorbidity index** | 1.29*** | [1.27, 1.31] | 1.26*** | [1.25, 1.28] | 1.11*** | [1.09, 1.12] |
| **IMD Income decile** |  |  |  |  |  |  |
| 1 † |  |  | 1 | [1.00, 1.00] | 1 | [1.00, 1.00] |
| 2 |  |  | 1.17*** | [1.16, 1.18] | 1.17*** | [1.16, 1.18] |
| 3 |  |  | 1.25*** | [1.24, 1.26] | 1.25*** | [1.24, 1.26] |
| 4 |  |  | 1.31*** | [1.30, 1.32] | 1.31*** | [1.30, 1.32] |
| 5 |  |  | 1.40*** | [1.39, 1.42] | 1.40*** | [1.39, 1.41] |
| 6 |  |  | 1.49*** | [1.48, 1.51] | 1.48*** | [1.47, 1.50] |
| 7 |  |  | 1.58*** | [1.56, 1.59] | 1.56*** | [1.55, 1.58] |
| 8 |  |  | 1.66*** | [1.64, 1.67] | 1.64*** | [1.63, 1.66] |
| 9 |  |  | 1.75*** | [1.74, 1.77] | 1.73*** | [1.72, 1.75] |
| 10-Most deprived |  |  | 1.80*** | [1.79, 1.82] | 1.77*** | [1.76, 1.79] |
| **Number of prior consultations** |  |  |  |  | 1.02*** | [1.02, 1.02] |
| Log Overdispersion parameter r | 4.21*** | [3.92, 4.51] | 12.59*** | [11.67, 13.58] | 12.16*** | [11.28, 13.11] |
| Log variance of the random effect | 18.73*** | [17.39, 20.16] | 61.62*** | [57.04, 66.56] | 59.16*** | [54.78, 63.89] |
| **Observations** | 11,406,264 | | 9,341,818 | | 9,241,818 | |

† Indicates the reference group. * = P <0.05, ** = P<0.01, *** = P <0.001

## Additional file 10 Risk factors and fit notes: Marginal effects from negative binomial regressions based on the pre COVID-19 pandemic cohort (2017-2019)

|  | **Margins** | **95% CI** |
| --- | --- | --- |
| **Sex** |  |  |
| Female† |  |  |
| Male | -0.33*** | [-0.33, -0.32] |
| **Age** |  |  |
| 18-30† |  |  |
| 31-40 | -0.05*** | [-0.06, -0.05] |
| 41-50 | 0.02*** | [0.02, 0.03] |
| 51-60 | 0.09*** | [0.08, 0.09] |
| 61-65 | -0.05*** | [-0.06, -0.05] |
| **Ethnicity** |  |  |
| White † |  |  |
| Asian | -0.25*** | [-0.26, -0.25] |
| Black | 0.17*** | [0.16, 0.18] |
| Missing | -0.21*** | [-0.22, -0.21] |
| Mixed | -0.37*** | [-0.39, -0.35] |
| Other | 0.05*** | [0.03, 0.06] |
| **BMI category** |  |  |
| Normal (18.5-24.9) † |  |  |
| Underweight (<18.5) | 0.08*** | [0.07, 0.09] |
| Overweight (25-29.9) | 0.14*** | [0.13, 0.14] |
| Obese (>=30) | 0.33*** | [0.33, 0.34] |
| Missing | -0.03** | [-0.03, -0.02] |
| **Region** |  |  |
| London † |  |  |
| East Midlands | 0.25*** | [0.23, 0.27] |
| East of England | 0.15*** | [0.14, 0.16] |
| Northeast | 0.30*** | [0.29, 0.31] |
| Northwest | 0.29*** | [0.29, 0.30] |
| Southeast | 0.19*** | [0.18, 0.20] |
| Southwest | 0.27*** | [0.26, 0.28] |
| West Midlands | 0.27*** | [0.26, 0.28] |
| Yorkshire and The Humber | 0.26*** | [0.25, 0.27] |
| **Smoking status** |  |  |
| Never smoked † |  |  |
| Ex-smoker | 0.22*** | [0.22, 0.23] |
| Current smoker | 0.42*** | [0.41, 0.42] |
| Missing | -0.26*** | [-0.27, -0.25] |
| **Comorbidity index** | 0.10*** | [0.08, 0.12] |
| **IMD Income decile** |  |  |
| Income decile=1 † |  |  |
| 2 | 0.16*** | [0.15, 0.17] |
| 3 | 0.22*** | [0.21, 0.23] |
| 4 | 0.27*** | [0.26, 0.28] |
| 5 | 0.34*** | [0.33, 0.34] |
| 6 | 0.39*** | [0.39, 0.40] |
| 7 | 0.45*** | [0.44, 0.45] |
| 8 | 0.50*** | [0.49, 0.50] |
| 9 | 0.55*** | [0.54, 0.56] |
| 10-Most deprived | 0.57*** | [0.56, 0.58] |
| **Number of prior consultations** | 0.02*** | [0.02, 0.02] |
| **Observations** | 9, 341, 818 |  |

† Indicates the reference group. * = P <0.05, ** = P<0.01, *** = P <0.001

## Additional file 11 Risk factors and fit notes: IRR from negative binomial regressions based on the late COVID-19 pandemic cohort (2022)

|  | **Model 1** | | **Model 2** | | **Model 3** | |
| --- | --- | --- | --- | --- | --- | --- |
|  | **IRR** | **95% CI** | **IRR** | **95% CI** | **IRR** | **95% CI** |
| **Sex** |  |  |  |  |  |  |
| Female † | 1 | [1.00, 1.00] | 1 | [1.00, 1.00] | 1 | [1.00, 1.00] |
| Male | 0.67*** | [0.67, 0.67] | 0.67*** | [0.66, 0.67] | 0.70*** | [0.70, 0.70] |
| **Age** |  |  |  |  |  |  |
| 18-30 † | 1 | [1.00, 1.00] | 1 | [1.00, 1.00] | 1 | [1.00, 1.00] |
| 31-40 | 0.97*** | [0.96, 0.98] | 0.96*** | [0.95, 0.97] | 0.96*** | [0.96, 0.97] |
| 41-50 | 1.00 | [0.99, 1.00] | 1.02*** | [1.01, 1.03] | 1.02*** | [1.01, 1.03] |
| 51-60 | 1.11*** | [1.10, 1.12] | 1.16*** | [1.15, 1.17] | 1.13*** | [1.12, 1.14] |
| 61-65 | 1.14*** | [1.13, 1.15] | 1.20*** | [1.19, 1.21] | 1.16*** | [1.15, 1.17] |
| **Ethnicity** |  |  |  |  |  |  |
| White † | 1 | [1.00, 1.00] | 1 | [1.00, 1.00] | 1 | [1.00, 1.00] |
| Asian | 0.95*** | [0.94, 0.95] | 0.91*** | [0.90, 0.92] | 0.91*** | [0.90, 0.92] |
| Black | 1.34*** | [1.32, 1.35] | 1.26*** | [1.25, 1.27] | 1.27*** | [1.25, 1.28] |
| Missing | 0.72*** | [0.71, 0.73] | 0.74*** | [0.73, 0.74] | 0.76*** | [0.75, 0.76] |
| Mixed | 0.80*** | [0.79, 0.82] | 0.73*** | [0.71, 0.75] | 0.75*** | [0.73, 0.77] |
| Other | 1.17*** | [1.15, 1.18] | 1.12*** | [1.10, 1.14] | 1.12*** | [1.10, 1.14] |
| **BMI category** |  |  |  |  |  |  |
| Normal (18.5-24.9) † | 1 | [1.00, 1.00] | 1 | [1.00, 1.00] | 1 | [1.00, 1.00] |
| Underweight (<18.5) | 1.12*** | [1.11, 1.14] | 1.12*** | [1.10, 1.14] | 1.11*** | [1.10, 1.13] |
| Overweight (25-29.9) | 1.19*** | [1.18, 1.19] | 1.18*** | [1.17, 1.19] | 1.17*** | [1.16, 1.17] |
| Obese (>=30) | 1.55*** | [1.54, 1.56] | 1.51*** | [1.50, 1.52] | 1.44*** | [1.44, 1.45] |
| Missing | 0.95*** | [0.94, 0.96] | 0.93*** | [0.92, 0.94] | 0.95*** | [0.94, 0.95] |
| **Region** |  |  |  |  |  |  |
| London † | 1 | [1.00, 1.00] | 1 | [1.00, 1.00] | 1 | [1.00, 1.00] |
| East Midlands | 1.22*** | [1.19, 1.25] | 1.29*** | [1.26, 1.33] | 1.35*** | [1.31, 1.39] |
| East of England | 0.97** | [0.95, 0.99] | 1.10*** | [1.08, 1.13] | 1.13*** | [1.10, 1.15] |
| Northeast | 1.54*** | [1.52, 1.57] | 1.54*** | [1.51, 1.57] | 1.51*** | [1.48, 1.54] |
| Northwest | 1.45*** | [1.44, 1.47] | 1.46*** | [1.44, 1.48] | 1.38*** | [1.37, 1.40] |
| Southeast | 1.08*** | [1.07, 1.09] | 1.25*** | [1.23, 1.26] | 1.23*** | [1.22, 1.25] |
| Southwest | 1.34*** | [1.32, 1.36] | 1.43*** | [1.41, 1.45] | 1.33*** | [1.31, 1.34] |
| West Midlands | 1.35*** | [1.33, 1.36] | 1.39*** | [1.37, 1.40] | 1.35*** | [1.34, 1.37] |
| Yorkshire and The Humber | 1.28*** | [1.25, 1.31] | 1.33*** | [1.30, 1.37] | 1.33*** | [1.30, 1.36] |
| **Smoking status** |  |  |  |  |  |  |
| Never smoked † | 1 | [1.00, 1.00] | 1 | [1.00, 1.00] | 1 | [1.00, 1.00] |
| Ex-smoker | 1.33*** | [1.32, 1.33] | 1.30*** | [1.30, 1.31] | 1.27*** | [1.27, 1.28] |
| Current smoker | 1.63*** | [1.62, 1.64] | 1.54*** | [1.53, 1.55] | 1.51*** | [1.50, 1.52] |
| Missing | 0.84*** | [0.83, 0.85] | 0.79*** | [0.78, 0.80] | 0.80*** | [0.78, 0.81] |
| **Comorbidity index** | 1.41*** | [1.38, 1.44] | 1.37*** | [1.34, 1.40] | 1.11*** | [1.08, 1.13] |
| **IMD Income decile** |  |  |  |  |  |  |
| Income decile=1 † |  |  | 1 | [1.00, 1.00] | 1 | [1.00, 1.00] |
| 2 |  |  | 1.21*** | [1.19, 1.23] | 1.21*** | [1.19, 1.23] |
| 3 |  |  | 1.29*** | [1.27, 1.30] | 1.28*** | [1.27, 1.30] |
| 4 |  |  | 1.36*** | [1.34, 1.38] | 1.36*** | [1.34, 1.38] |
| 5 |  |  | 1.47*** | [1.45, 1.49] | 1.46*** | [1.44, 1.48] |
| 6 |  |  | 1.57*** | [1.55, 1.59] | 1.56*** | [1.54, 1.58] |
| 7 |  |  | 1.66*** | [1.64, 1.68] | 1.64*** | [1.62, 1.66] |
| 8 |  |  | 1.77*** | [1.74, 1.79] | 1.75*** | [1.72, 1.77] |
| 9 |  |  | 1.86*** | [1.84, 1.89] | 1.84*** | [1.81, 1.86] |
| 10-Most deprived |  |  | 1.93*** | [1.91, 1.96] | 1.89*** | [1.87, 1.92] |
| **Number of prior consultations** |  |  |  |  | 1.02*** | [1.02, 1.02] |
| Overdispersion parameter r | 11.91*** | [11.08, 12.79] | 16.68*** | [15.36, 18.12] | 13.07*** | [12.06, 14.17] |
| The variance of the random effect | 37.32*** | [34.69, 40.15] | 53.66*** | [49.34, 58.36] | 41.52*** | [38.26, 45.07] |
| **Observations** | 10,044,331 |  | 7,520,270 |  | 7,520,270 |  |

† Indicates the reference group. * = P <0.05, ** = P<0.01, *** = P <0.001

## Additional file 12 Risk factors and fit notes: Marginal effects from negative binomial regressions based on the late COVID-19 pandemic cohort (2022)

|  | **Margins** | **95% CI** |
| --- | --- | --- |
| **Sex** |  |  |
| Female † |  |  |
| Male | -0.36*** | [-0.36, -0.35] |
| **Age** |  |  |
| 18-30 † |  |  |
| 31-40 | -0.04*** | [-0.05, -0.03] |
| 41-50 | 0.02*** | [0.01, 0.03] |
| 51-60 | 0.12*** | [0.12, 0.13] |
| 61-65 | 0.15*** | [0.14, 0.16] |
| **Ethnicity** |  |  |
| White † |  |  |
| Asian | -0.09*** | [-0.10, -0.09] |
| Black | 0.24*** | [0.23, 0.25] |
| Missing | -0.28*** | [-0.29, -0.27] |
| Mixed | -0.29*** | [-0.32, -0.26] |
| Other | 0.12*** | [0.10, 0.13] |
| **BMI category** |  |  |
| Normal (18.5-24.9) † |  |  |
| Underweight (<18.5) | 0.11*** | [0.10, 0.13] |
| Overweight (25-29.9) | 0.15*** | [0.15, 0.16] |
| Obese (>=30) | 0.37*** | [0.36, 0.38] |
| Missing | -0.06*** | [-0.07, -0.05] |
| **Region** |  |  |
| London † |  |  |
| East Midlands | 0.30*** | [0.27, 0.33] |
| East of England | 0.12*** | [0.10, 0.14] |
| Northeast | 0.41*** | [0.39, 0.43] |
| Northwest | 0.32*** | [0.31, 0.34] |
| Southeast | 0.21*** | [0.20, 0.22] |
| Southwest | 0.28*** | [0.27, 0.30] |
| West Midlands | 0.30*** | [0.29, 0.31] |
| Yorkshire and The Humber | 0.28*** | [0.26, 0.31] |
| **Smoking status** |  |  |
| Never smoked † |  |  |
| Ex-smoker | 0.24*** | [0.24, 0.25] |
| Current smoker | 0.41*** | [0.41, 0.42] |
| Missing | -0.22*** | [-0.23, -0.20] |
| **Comorbidity index** | 0.10*** | [0.08, 0.12] |
| **IMD Income decile** |  |  |
| Income decile=1 † |  |  |
| 2 | 0.19*** | [0.18, 0.20] |
| 3 | 0.25*** | [0.23, 0.26] |
| 4 | 0.30*** | [0.29, 0.32] |
| 5 | 0.38*** | [0.36, 0.39] |
| 6 | 0.44*** | [0.43, 0.46] |
| 7 | 0.49*** | [0.48, 0.51] |
| 8 | 0.56*** | [0.54, 0.57] |
| 9 | 0.61*** | [0.59, 0.62] |
| 10-Most deprived | 0.64*** | [0.62, 0.65] |
| **Number of prior consultations** | 0.02*** | [0.02, 0.02] |
| **Observations** | 7, 520, 270 |  |

† Indicates the reference group. * = P <0.05, ** = P<0.01, *** = P <0.001

## Additional file 13 Risk factors and fit notes: IRR from fixed-effect Poisson regressions based on the pre COVID-19 pandemic cohort (2017-2019)

|  | Model 1 | | Model 2 | | Model 3 | |
| --- | --- | --- | --- | --- | --- | --- |
|  | IRR | 95% CI | IRR | 95% CI | IRR | 95% CI |
| **Sex** |  |  |  |  |  |  |
| Female † | 1 | [1.00,1.00] | 1 | [1.00,1.00] | 1 | [1.00,1.00] |
| Male | 0.73*** | [0.72,0.74] | 0.73*** | [0.72,0.73] | 0.75*** | [0.75,0.76] |
| **Age** |  |  |  |  |  |  |
| 18-30 † | 1 | [1.00,1.00] | 1 | [1.00,1.00] | 1 | [1.00,1.00] |
| 31-40 | 1.03*** | [1.02,1.05] | 1.04*** | [1.02,1.06] | 1.04*** | [1.02,1.05] |
| 41-50 | 1.16*** | [1.14,1.18] | 1.19*** | [1.17,1.21] | 1.18*** | [1.16,1.20] |
| 51-60 | 1.30*** | [1.27,1.32] | 1.34*** | [1.31,1.36] | 1.30*** | [1.28,1.33] |
| 61-65 | 1.18*** | [1.15,1.20] | 1.23*** | [1.20,1.25] | 1.17*** | [1.14,1.19] |
| **Ethnicity** |  |  |  |  |  |  |
| White † |  |  |  |  |  |  |
| Asian | 0.81*** | [0.79,0.83] | 0.80*** | [0.78,0.83] | 0.80*** | [0.77,0.82] |
| Black | 1.20*** | [1.17,1.24] | 1.18*** | [1.14,1.21] | 1.18*** | [1.14,1.22] |
| Missing | 0.78*** | [0.77,0.80] | 0.78*** | [0.77,0.80] | 0.80*** | [0.78,0.81] |
| Mixed | 0.68*** | [0.64,0.73] | 0.69*** | [0.66,0.73] | 0.70*** | [0.67,0.74] |
| Other | 1.09*** | [1.06,1.12] | 1.08*** | [1.05,1.11] | 1.08*** | [1.06,1.11] |
| **BMI** |  |  |  |  |  |  |
| Normal (18.5-24.9) † | 1 | [1.00,1.00] | 1 | [1.00,1.00] | 1 | [1.00,1.00] |
| Underweight (<18.5) | 1.16*** | [1.14,1.17] | 1.14*** | [1.13,1.16] | 1.13*** | [1.12,1.15] |
| Overweight (25-29.9) | 1.14*** | [1.13,1.15] | 1.14*** | [1.13,1.14] | 1.13*** | [1.12,1.14] |
| Obese (>=30) | 1.48*** | [1.46,1.49] | 1.45*** | [1.44,1.47] | 1.40*** | [1.39,1.42] |
| Missing | 0.96*** | [0.95,0.97] | 0.96*** | [0.94,0.97] | 0.97*** | [0.96,0.98] |
| **Smoking status** |  |  |  |  |  |  |
| Never smoked † | 1 | [1.00,1.00] | 1 | [1.00,1.00] | 1 | [1.00,1.00] |

|  | Model 1 | | Model 2 | | Model 3 | |
| --- | --- | --- | --- | --- | --- | --- |
|  | IRR | 95% CI | IRR | 95% CI | IRR | 95% CI |
| Ex-smoker | 1.32*** | [1.31,1.33] | 1.31*** | [1.30,1.32] | 1.28*** | [1.27,1.29] |
| Current smoker | 1.80*** | [1.78,1.82] | 1.72*** | [1.70,1.74] | 1.70*** | [1.68,1.71] |
| Missing | 0.72*** | [0.70,0.75] | 0.73*** | [0.71,0.75] | 0.73*** | [0.72,0.75] |
| **Comorbidity index** | 1.36*** | [1.33,1.39] | 1.34*** | [1.31,1.37] | 1.12*** | [1.08,1.17] |
| **IMD Income decile** |  |  |  |  |  |  |
| 1 † |  |  | 1 | [1.00,1.00] | 1 | [1.00,1.00] |
| 2 |  |  | 1.17*** | [1.14,1.21] | 1.17*** | [1.14,1.20] |
| 3 |  |  | 1.27*** | [1.24,1.31] | 1.26*** | [1.23,1.30] |
| 4 |  |  | 1.37*** | [1.33,1.40] | 1.35*** | [1.32,1.39] |
| 5 |  |  | 1.45*** | [1.42,1.49] | 1.44*** | [1.40,1.47] |
| 6 |  |  | 1.58*** | [1.53,1.62] | 1.56*** | [1.51,1.60] |
| 7 |  |  | 1.68*** | [1.63,1.73] | 1.65*** | [1.60,1.70] |
| 8 |  |  | 1.79*** | [1.73,1.84] | 1.75*** | [1.70,1.80] |
| 9 |  |  | 1.92*** | [1.86,1.98] | 1.88*** | [1.82,1.93] |
| 10-Most deprived |  |  | 2.03*** | [1.96,2.10] | 1.97*** | [1.91,2.03] |
| **Number of prior consultations** |  |  |  |  | 1.02*** | [1.02,1.02] |
| **Observations** | 11,373,295 |  | 9,341,818 |  | 9,341,818 |  |

† Indicates the reference group. * = P <0.05, ** = P<0.01, *** = P <0.001

Note: As noted by Allison and Waterman [5], although fixed-effects negative binomial models are intended to adjust for unobserved time-invariant heterogeneity, the fixed-effects estimators implemented in standard statistical software (e.g., Stata) do not fully condition out all time-invariant variables. Given this limitation, estimates from fixed-effects Poisson regression models are reported here for the sensitivity analysis, which offer more robust inference in this setting [5].

## Additional file 14 Risk factors and fit notes: IRR from fixed-effect Poisson regressions based on the late COVID-19 pandemic cohort (2022)

|  | **Model 1** | | **Model 2** | | **Model 3** | |
| --- | --- | --- | --- | --- | --- | --- |
|  | **IRR** | **95% CI** | **IRR** | **95% CI** | **IRR** | **95% CI** |
| **Sex** |  |  |  |  |  |  |
| Female † | 1 | [1.00,1.00] | 1 | [1.00,1.00] | 1 | [1.00,1.00] |
| Male | 0.69*** | [0.68,0.70] | 0.69*** | [0.68,0.70] | 0.73*** | [0.72,0.74] |
| **Age** |  |  |  |  |  |  |
| 18-30 † | 1 | [1.00,1.00] | 1 | [1.00,1.00] | 1 | [1.00,1.00] |
| 31-40 | 1.04*** | [1.02,1.06] | 1.03** | [1.01,1.05] | 1.03*** | [1.01,1.05] |
| 41-50 | 1.12*** | [1.10,1.15] | 1.14*** | [1.12,1.16] | 1.13*** | [1.11,1.15] |
| 51-60 | 1.29*** | [1.26,1.32] | 1.32*** | [1.30,1.35] | 1.29*** | [1.26,1.31] |
| 61-65 | 1.40*** | [1.37,1.43] | 1.45*** | [1.42,1.48] | 1.39*** | [1.36,1.42] |
| **Ethnicity** |  |  |  |  |  |  |
| White † | 1 | [1.00,1.00] | 1 | [1.00,1.00] | 1 | [1.00,1.00] |
| Asian | 0.91*** | [0.89,0.94] | 0.91*** | [0.88,0.93] | 0.90*** | [0.88,0.93] |
| Black | 1.25*** | [1.21,1.29] | 1.22*** | [1.18,1.26] | 1.22*** | [1.18,1.26] |
| Missing | 0.69*** | [0.67,0.71] | 0.71*** | [0.69,0.72] | 0.73*** | [0.71,0.75] |
| Mixed | 0.82*** | [0.76,0.89] | 0.77*** | [0.73,0.81] | 0.78*** | [0.75,0.82] |
| Other | 1.16*** | [1.12,1.19] | 1.13*** | [1.09,1.16] | 1.13*** | [1.09,1.16] |
| **BMI** |  |  |  |  |  |  |
| Normal (18.5-24.9) † | 1 | [1.00,1.00] | 1 | [1.00,1.00] | 1 | [1.00,1.00] |
| Underweight (<18.5) | 1.16*** | [1.14,1.18] | 1.16*** | [1.13,1.18] | 1.14*** | [1.12,1.17] |
| Overweight (25-29.9) | 1.17*** | [1.16,1.18] | 1.17*** | [1.15,1.18] | 1.15*** | [1.14,1.16] |
| Obese (>=30) | 1.54*** | [1.52,1.56] | 1.52*** | [1.50,1.54] | 1.43*** | [1.41,1.45] |
| Missing | 0.95*** | [0.94,0.97] | 0.93*** | [0.91,0.94] | 0.95*** | [0.93,0.96] |
| **Smoking status** |  |  |  |  |  |  |
| Never smoked † | 1 | [1.00,1.00] | 1 | [1.00,1.00] | 1 | [1.00,1.00] |

|  | **Model 1** | | **Model 2** | | **Model 3** | |
| --- | --- | --- | --- | --- | --- | --- |
|  | IRR | 95% CI | IRR | 95% CI | IRR | 95% CI |
| Ex-smoker | 1.35*** | [1.34,1.36] | 1.33*** | [1.32,1.35] | 1.29*** | [1.28,1.31] |
| Current smoker | 1.73*** | [1.71,1.75] | 1.65*** | [1.63,1.68] | 1.61*** | [1.59,1.63] |
| Missing | 0.80*** | [0.76,0.84] | 0.76*** | [0.74,0.78] | 0.76*** | [0.74,0.78] |
| **Comorbidity index** | 1.45*** | [1.41,1.48] | 1.42*** | [1.38,1.46] | 1.07** | [1.02,1.13] |
| **IMD Income decile** |  |  |  |  |  |  |
| 1 † |  |  | 1 | [1.00,1.00] | 1 | [1.00,1.00] |
| 2 |  |  | 1.20*** | [1.16,1.24] | 1.18*** | [1.15,1.22] |
| 3 |  |  | 1.29*** | [1.25,1.33] | 1.27*** | [1.23,1.31] |
| 4 |  |  | 1.38*** | [1.33,1.42] | 1.35*** | [1.31,1.39] |
| 5 |  |  | 1.48*** | [1.43,1.53] | 1.44*** | [1.40,1.49] |
| 6 |  |  | 1.59*** | [1.54,1.65] | 1.55*** | [1.50,1.60] |
| 7 |  |  | 1.69*** | [1.63,1.75] | 1.64*** | [1.59,1.69] |
| 8 |  |  | 1.81*** | [1.75,1.87] | 1.74*** | [1.69,1.80] |
| 9 |  |  | 1.92*** | [1.85,1.99] | 1.84*** | [1.78,1.90] |
| 10-Most deprived |  |  | 2.02*** | [1.95,2.10] | 1.93*** | [1.86,1.99] |
| **Number of prior consultations** | |  |  |  | 1.02*** | [1.02,1.03] |
| **Observations** | 10,043,701 |  | 7,519,632 |  | 7,519,632 |  |

† Indicates the reference group. * = P <0.05, ** = P<0.01, *** = P <0.001

Note: As noted by Allison and Waterman [5], although fixed-effects negative binomial models are intended to adjust for unobserved time-invariant heterogeneity, the fixed-effects estimators implemented in standard statistical software (e.g., Stata) do not fully condition out all time-invariant variables. Given this limitation, estimates from fixed-effects Poisson regression models are reported here for the sensitivity analysis, which offer more robust inference in this setting [5].

## Additional file 15 Average cost estimation for English nations in each cohort

Additional file 15 (1) Average cost estimation for English nations in 2017 and 2018

| **Year** | **2017** | | | **2018** | | |
| --- | --- | --- | --- | --- | --- | --- |
|  | **Mean (£)** | **95% CIs** | | **Mean (£)** | **95% CIs** | |
| Northeast | 355 | 340 | 362 | 343 | 336 | 349 |
| Northwest | 403 | 399 | 406 | 389 | 386 | 393 |
| Yorkshire and The Humber | 393 | 385 | 400 | 371 | 364 | 379 |
| East Midlands | 384 | 375 | 394 | 370 | 362 | 379 |
| West Midlands | 398 | 394 | 402 | 382 | 379 | 385 |
| East of England | 457 | 449 | 465 | 432 | 424 | 440 |
| London | 259 | 257 | 261 | 246 | 244 | 248 |
| Southeast | 424 | 420 | 427 | 410 | 406 | 413 |
| Southwest | 429 | 424 | 433 | 416 | 411 | 420 |
| England | 378 | 377 | 380 | 363 | 362 | 364 |

Note: Multiple assumptions and sources used for cost modelling, therefore individual trends may not fully triangulate with final cost numbers.

Additional file 15 (2) Average cost estimation for English nations in 2019 and 2022

| **Year** | **2019** | | | **2022** | | |
| --- | --- | --- | --- | --- | --- | --- |
|  | **Mean (£)** | **95% CIs** | | **Mean (£)** | **95% CIs** | |
| Northeast | 355 | 349 | 362 | 436 | 427 | 445 |
| Northwest | 416 | 412 | 419 | 508 | 504 | 513 |
| Yorkshire and The Humber | 362 | 355 | 369 | 383 | 373 | 393 |
| East Midlands | 333 | 325 | 341 | 350 | 340 | 361 |
| West Midlands | 408 | 404 | 411 | 511 | 506 | 516 |
| East of England | 449 | 441 | 458 | 541 | 530 | 552 |
| London | 256 | 254 | 259 | 318 | 315 | 321 |
| Southeast | 436 | 432 | 439 | 504 | 499 | 508 |
| Southwest | 431 | 427 | 436 | 468 | 463 | 473 |
| England | 380 | 378 | 381 | 451 | 449 | 453 |

Note: Multiple assumptions and sources used for cost modelling, therefore individual trends may not fully triangulate with final cost numbers.

## Additional file 16 Estimated regional total cost of sickness absence

Additional file 16 (1) Estimated regional total cost of sickness absence (in 2017 and 2018)

| **Year** | **2017** | | | **2018** | | |
| --- | --- | --- | --- | --- | --- | --- |
|  | **Mean (£)** | **95% CIs** | | **Mean (£)** | **95% CIs** | |
| Northeast | £0.58bn | £0.57bn | £0.59bn | £0.56bn | £0.55bn | £0.57bn |
| Northwest | £1.85bn | £1.84bn | £1.87bn | £1.79bn | £1.78bn | £1.81bn |
| Yorkshire and The Humber | £1.33bn | £1.30bn | £1.35bn | £1.26bn | £1.23bn | £1.28bn |
| East Midlands | £1.16bn | £1.13bn | £1.19bn | £1.12bn | £1.09bn | £1.14bn |
| West Midlands | £1.45bn | £1.43bn | £1.46bn | £1.39bn | £1.38bn | £1.40bn |
| East of England | £1.76bn | £1.73bn | £1.79bn | £1.66bn | £1.63bn | £1.69bn |
| London | £1.55bn | £1.53bn | £1.56bn | £1.47bn | £1.46bn | £1.48bn |
| Southeast | £2.40bn | £2.38bn | £2.42bn | £2.32bn | £2.30bn | £2.34bn |
| Southwest | £1.47bn | £1.46bn | £1.49bn | £1.43bn | £1.41bn | £1.44bn |
| England | £13.31bn | £13.25bn | £13.36bn | £12.77bn | £12.73bn | £12.82bn |

Note: Multiple assumptions and sources used for cost modelling, therefore individual trends may not fully triangulate with final cost numbers.

Additional file 16 (2) Estimated regional total cost of sickness absence (in 2019 and 2022)

|  | **2019** | | | **2022** | | |
| --- | --- | --- | --- | --- | --- | --- |
|  | **Mean (£)** | **95% CIs** | | **Mean (£)** | **95% CIs** | |
| Northeast | £0.58bn | £0.57bn | £0.59bn | £0.71bn | £0.70bn | £0.73bn |
| Northwest | £1.91bn | £1.90bn | £1.93bn | £2.34bn | £2.32bn | £2.36bn |
| Yorkshire and The Humber | £1.22bn | £1.20bn | £1.25bn | £1.30bn | £1.26bn | £1.33bn |
| East Midlands | £1.00bn | £0.98bn | £1.03bn | £1.06bn | £1.02bn | £1.09bn |
| West Midlands | £1.48bn | £1.47bn | £1.50bn | £1.86bn | £1.84bn | £1.88bn |
| East of England | £1.73bn | £1.70bn | £1.76bn | £2.08bn | £2.04bn | £2.12bn |
| London | £1.53bn | £1.52bn | £1.54bn | £1.90bn | £1.88bn | £1.92bn |
| Southeast | £2.47bn | £2.45bn | £2.49bn | £2.86bn | £2.83bn | £2.88bn |
| Southwest | £1.48bn | £1.47bn | £1.50bn | £1.61bn | £1.59bn | £1.63bn |
| England | £13.36bn | £13.31bn | £13.41bn | £15.87bn | £15.80bn | £15.93bn |

Note: Multiple assumptions and sources used for cost modelling, therefore individual trends may not fully triangulate with final cost numbers.

## Additional file 17 Sensitivity analysis: Average cost estimation for English nations in each cohort

| **Year** | **2017** | | | **2018** | | | **2019** | | | **2022** | | |
| --- | --- | --- | --- | --- | --- | --- | --- | --- | --- | --- | --- | --- |
|  | **Mean (£)** | **95% CIs** | | **Mean (£)** | **95% CIs** | | **Mean (£)** | **95% CIs** | | **Mean (£)** | **95% CIs** | |
| Northeast | 433 | 425 | 441 | 418 | 410 | 425 | 434 | 426 | 441 | 532 | 522 | 543 |
| Northwest | 463 | 459 | 467 | 444 | 441 | 448 | 475 | 471 | 479 | 581 | 576 | 586 |
| Yorkshire and The Humber | 393 | 385 | 400 | 371 | 364 | 379 | 362 | 355 | 369 | 383 | 373 | 393 |
| East Midlands | 410 | 401 | 420 | 397 | 387 | 406 | 357 | 348 | 366 | 389 | 377 | 401 |
| West Midlands | 467 | 463 | 471 | 450 | 446 | 454 | 482 | 478 | 486 | 606 | 600 | 611 |
| East of England | 481 | 472 | 490 | 459 | 450 | 468 | 464 | 455 | 473 | 564 | 552 | 576 |
| London | 337 | 334 | 340 | 321 | 318 | 324 | 334 | 331 | 337 | 416 | 413 | 420 |
| Southeast | 425 | 421 | 429 | 410 | 407 | 413 | 435 | 432 | 439 | 502 | 497 | 506 |
| Southwest | 458 | 453 | 463 | 446 | 441 | 450 | 460 | 456 | 465 | 497 | 491 | 502 |
| England | 424 | 422 | 426 | 407 | 406 | 409 | 426 | 424 | 427 | 509 | 507 | 511 |

*Note:* Multiple assumptions and sources used for cost modelling, therefore individual trends may not fully triangulate with final cost numbers

## Additional file 18 Sensitivity analysis: Regional total cost of sickness absence

Additional file 18 (1) Sensitivity analysis: Regional total cost of sickness absence (in 2017 and 2018)

| **Year** | **2017** | | | **2018** | | |
| --- | --- | --- | --- | --- | --- | --- |
|  | **Mean (£)** | **95% CIs** | | **Mean (£)** | **95% CIs** | |
| Northeast | £0.71bn | £0.69bn | £0.72bn | £0.68bn | £0.67bn | £0.69bn |
| Northwest | £2.13bn | £2.11bn | £2.15bn | £2.05bn | £2.03bn | £2.06bn |
| Yorkshire and The Humber | £1.33bn | £1.30bn | £1.35bn | £1.26bn | £1.23bn | £1.28bn |
| East Midlands | £1.24bn | £1.21bn | £1.27bn | £1.20bn | £1.17bn | £1.22bn |
| West Midlands | £1.70bn | £1.68bn | £1.71bn | £1.64bn | £1.62bn | £1.65bn |
| East of England | £1.85bn | £1.82bn | £1.89bn | £1.77bn | £1.73bn | £1.80bn |
| London | £2.01bn | £2.00bn | £2.03bn | £1.92bn | £1.90bn | £1.93bn |
| Southeast | £2.41bn | £2.39bn | £2.43bn | £2.33bn | £2.31bn | £2.34bn |
| Southwest | £1.57bn | £1.56bn | £1.59bn | £1.53bn | £1.52bn | £1.55bn |
| England | £14.92bn | £14.87bn | £14.98bn | £14.33bn | £14.28bn | £14.38bn |

*Note:* Multiple assumptions and sources used for cost modelling, therefore individual trends may not fully triangulate with final cost numbers

Additional file 18 (2) Sensitivity analysis: Regional total cost of sickness absence (in 2019 and 2022)

| **Year** | **2019** | | | **2022** | | |
| --- | --- | --- | --- | --- | --- | --- |
|  | **Mean (£)** | **95% CIs** | | **Mean (£)** | **95% CIs** | |
| Northeast | £0.71bn | £0.69bn | £0.72bn | £0.87bn | £0.85bn | £0.89bn |
| Northwest | £2.18bn | £2.17bn | £2.20bn | £2.67bn | £2.65bn | £2.70bn |
| Yorkshire and The Humber | £1.22bn | £1.20bn | £1.25bn | £1.30bn | £1.26bn | £1.33bn |
| East Midlands | £1.08bn | £1.05bn | £1.10bn | £1.17bn | £1.14bn | £1.21bn |
| West Midlands | £1.75bn | £1.74bn | £1.77bn | £2.20bn | £2.18bn | £2.22bn |
| East of England | £1.79bn | £1.75bn | £1.82bn | £2.17bn | £2.12bn | £2.22bn |
| London | £1.99bn | £1.97bn | £2.01bn | £2.49bn | £2.46bn | £2.51bn |
| Southeast | £2.47bn | £2.45bn | £2.49bn | £2.85bn | £2.82bn | £2.87bn |
| Southwest | £1.58bn | £1.57bn | £1.60bn | £1.71bn | £1.69bn | £1.73bn |
| England | £14.98bn | £14.92bn | £15.03bn | £17.92bn | £17.85bn | £17.99bn |

*Note:* Multiple assumptions and sources used for cost modelling, therefore individual trends may not fully triangulate with final cost numbers

## Additional file 19 Medical code lists used for fit note (“not fit for work”)

| **MEDICAL_CODE_ID** | **DESCRIPTION** | **SNOMED_CT_CODE** |
| --- | --- | --- |
| 284745019 | Private sickness cert. NOS | 307905007 |
| 8006461000006110 | Sickness notification of general practitioner | 18691000000103 |
| 12451431000006100 | Sickness notification-of GP | 18691000000103 |
| 12487881000006100 | MED3 issued to patient | 1331000000103 |
| 8006481000006110 | Sickness notification of GP (general practitioner) | 18691000000103 |
| 7968061000006110 | Med3 certificate issued to patient | 1331000000103 |
| 2168601000000110 | Sickness certificate | 18381000000107 |
| 11661000000116 | MED5 - NOS | 5131000000103 |
| 1842241000006110 | MED3 (2010) - expected duration unfit for work | 1842241000006100 |
| 11641000000117 | MED5 - issued to patient | 1371000000101 |
| 11631000000114 | MED5 issued to patient | 1371000000101 |
| 11591000000114 | MED3 - NOS | 5121000000100 |
| 1653351000000110 | eMED3 (2010) new statement issued not fit for work | 751481000000104 |
| 11561000000115 | MED3 issued to patient | 1331000000103 |
| 30221000000112 | Sickness certificates | 18381000000107 |
| 1653921000000110 | MED3 (2010) issued by hand not fit for work | 751731000000106 |
| 11701000000110 | Private sickness certificate | 1411000000102 |
| 11551000000118 | MED3 - doctor's statement | 1321000000100 |
| 11621000000112 | MED5 - doctor's special stat. | 1361000000108 |
| 32221000000111 | Sickness notification-of GP | 18691000000103 |
| 1769621000006110 | MED3 (2010) certificate issued to patient | 775241000000108 |
| 226031000000112 | Sick note generated from secondary care done by practice | 165801000000106 |
| 1817471000006110 | Sickness certificates | 18381000000107 |

## Additional file 20 Patient and public involvement (PPIE)

Patients and members of the public were first involved during the dissemination and interpretation phase of this study. The study findings were presented to and discussed with a group of patients with experience of having long-term health conditions. PPIE members discussed the importance of understanding the link between health and employment, considering changes before and after COVID-19, the practical use of existing data, and the wider implications of the findings. These informed the interpretation of results and enhanced the real-world relevance of the final manuscript.

## References

1. Office for National Statistics. Admin‑based income statistics, England and Wales: tax year ending 2018 [Experimental Statistics]. London: Office for National Statistics; 2022 Dec 20. Available from: Admin‑based income statistics, England and Wales: tax year ending 2018. Available from: <https://www.ons.gov.uk/peoplepopulationandcommunity/personalandhouseholdfinances/incomeandwealth/articles/adminbasedincomestatisticsenglandandwales/taxyearending2018>
2. Office for National Statistics. Research outputs: Income from Pay As You Earn (PAYE) and benefits for tax year ending 2016 [Internet]. Census Transformation Programme. Available from: <https://www.ons.gov.uk/census/censustransformationprogramme/administrativedatacensusproject/administrativedatacensusresearchoutputs/populationcharacteristics/researchoutputsincomefrompayasyouearnpayeandbenefitsfortaxyearending2016>
3. Office for National Statistics. Dataset EARN01: Average weekly earnings [Internet]. London: Office for National Statistics; 17 July 2025. Available from: <https://www.ons.gov.uk/employmentandlabourmarket/peopleinwork/earningsandworkinghours/datasets/averageweeklyearningsearn01> [dera.ioe.ac.uk+12ons.gov.uk+12uksa.statisticsauthority.gov.uk+12](https://www.ons.gov.uk/employmentandlabourmarket/peopleinwork/earningsandworkinghours/datasets/averageweeklyearningsearn01?utm_source=chatgpt.com)
4. NHS Digital. Fit Notes Issued by GP Practices, England [statistical series]. London: NHS Digital; 10 April 2025. Available from: <https://digital.nhs.uk/data-and-information/publications/statistical/fit-notes-issued-by-gp-practices>.
5. Allison PD, Waterman RP. Fixed-effects negative binomial regression models. Sociol Methodol. 2002;32(1):247–65.
